# Genome-Wide Characterization of the Methyl CpG Binding Domain-Containing Proteins in Watermelon and Functional Analysis of Their Roles in Disease Resistance Through Ectopic Overexpression in *Arabidopsis thaliana*

**DOI:** 10.3389/fpls.2022.886965

**Published:** 2022-05-09

**Authors:** Jiayu Liang, Xiaodan Li, Ya Wen, Xinyi Wu, Hui Wang, Dayong Li, Fengming Song

**Affiliations:** Zhejiang Provincial Key Laboratory of Biology of Crop Pathogens and Insects, Ministry of Agriculture and Rural Affairs (MARA) Key Laboratory of Molecular Biology of Crop Pathogens and Insects, College of Agriculture and Biotechnology, Institute of Biotechnology, Zhejiang University, Hangzhou, China

**Keywords:** watermelon (*Citrullus lanatus* L.), methyl-CPG-binding domain (MBD) protein, *ClMBD2*, disease resistance, DNA methylation, Arabidopsis

## Abstract

Methyl-CPG-Binding Domain (MBD) proteins play important roles in plant growth, development, and stress responses. The present study characterized the *MBD* families in watermelon and other cucurbit plants regarding the gene numbers and structures, phylogenetic and syntenic relationships, evolution events, and conserved domain organization of the MBD proteins. The watermelon ClMBD proteins were found to be localized in nucleus, and ClMBD2 and ClMBD3 interacted with ClIDM2 and ClIDM3. ClMBD2 bound to DNA harboring methylated CG sites but not to DNA with methylated CHG and CHH sites *in vitro*. The *ClMBD* genes exhibited distinct expression patterns in watermelon plants after SA and MeJA treatment and after infection by fungal pathogens *Fusarium oxysporum* f.sp. *niveum* and *Didymella bryoniae*. Overexpression of *ClMBD2*, *ClMBD3*, or *ClMBD5* in Arabidopsis resulted in attenuated resistance against *Botrytis cinerea*, accompanied by down-regulated expression of *AtPDF1.2* and increased accumulation of H_2_O_2_ upon *B. cinerea* infection. Overexpression of *ClMBD1* and *ClMBD2* led to down-regulated expression of *AtPR1* and decreased resistance while overexpression of *ClMBD5* resulted in up-regulated expression of *AtPR1* and increased resistance against *Pseudomonas syringae* pv. *tomato* DC3000. Transcriptome analysis revealed that overexpression of *ClMBD2* in Arabidopsis up-regulated the expression of a small set of genes that negatively regulate Arabidopsis immunity. These data suggest the importance of some *ClMBD* genes in plant immunity and provide the possibility to improve plant immunity through modification of specific *ClMBD* genes.

## Introduction

As sessile organisms, plants have to face invasive attacks from diverse pathogenic microorganisms in the environment. To defense these pathogenic invasions, plants have evolved a complicated but fine-tuned innate immune system (Jones and Dangl, 2006; Yuan et al., 2021; Ngou et al., 2022). The first layer of the innate immunity, called PAMP-triggered immunity (PTI), is triggered by the recognition of microbial patterns via cell surface-localized pattern-recognition receptors, while the second layer, called effector-triggered immunity (ETI), is activated by the direct or indirect interaction between predominantly intracellularly localized nucleotide-binding leucine-rich repeat receptors and pathogen effectors. Activation of PTI and/or ETI is fine-tuned by a complicated molecular and genetic network, in which epigenetic regulation including DNA methylation/demethylation play critical roles (Huang and Jin, 2022).

Methylation of DNA, a conserved epigenetic mark, is one of the main mechanisms that play critical roles in epigenetic regulation of various biological processes including plant growth, development, and response to environmental cues (He et al., 2011; Arıkan et al., 2018; Zhang et al., 2018). Among DNA methylation, cytosine methylation (5-mC) is the most common epigenetic phenomenon that regulates the fate of gene expression (Grafi et al., 2007; Zhang et al., 2018). In higher plants, 5-mC occurs in CG dinucleotide regions, and CHG/CHH (H represents A, T, or C) trinucleotide regions (Gruenbaum et al., 1981). DNA methylation is a dynamic process that are achieved by different enzymes (Moore et al., 2013; Zhang et al., 2018) and is involved in many molecular processes, including genome stability, gene regulation, transposon silencing, and chromosome interactions (Zhang et al., 2006; Cokus et al., 2008; Lang et al., 2017). Extensive studies have indicated that DNA methylation plays important roles in plant growth and development, such as vegetable growth, pattern formation, flowering time, seed development, and fruit ripening (Gehring et al., 2009; He et al., 2011; Ibarra et al., 2012; Arıkan et al., 2018; Zhang et al., 2018) as well as in abiotic stress responses (Rambani et al., 2015; Xu et al., 2015; Yong-Villalobos et al., 2015; Hewezi et al., 2017). Importantly, DNA methylation, as one of the epigenetic regulation mechanisms, plays crucial roles in plant immunity (Huang and Jin, 2022); for example, Arabidopsis mutants with DNA hypomethylation are more resistant to *Pseudomonas syringae* pv. *tomato* (*Pst*) DC3000 and exhibit an elevated salicylic acid (SA)-dependent response (Dowen et al., 2012; Yu et al., 2013; Cambiagno et al., 2021).

In epigenetic model, proteins of the Methyl-CpG-binding domain (MBD) family are a group of key interpreters of DNA methylation and are generally associated with transcriptional silencing (Law and Jacobsen, 2010; He et al., 2011). The MBD proteins typically contain an MBD domain, with the ability to bind to 5-mC DNA (Ohki et al., 2001; Zemach and Grafi, 2003). Generally, the MBD proteins recognize 5-mC and recruit histone deacetylases, chromatin remodelers, and histone methyltransferases to repress transcription (Gigek et al., 2016). Genes coding for MBD proteins have been characterized in some plant species including Arabidopsis, rice, maize, poplar, potato, tomato, petunia, common bean, soybean, and rapeseed (Grafi et al., 2007; Parida et al., 2018; Coelho et al., 2022; Shi et al., 2022; Xiao et al., 2022); for example, 13 *AtMBDs* in Arabidopsis, 17 *OsMBDs* in rice, 14 *ZmMBDs* in maize, and 14 *PtMBDs* in poplar were identified (Grafi et al., 2007). The Arabidopsis AtMBD proteins can be divided into different subclasses (Zemach and Grafi, 2003; Springer and Kaeppler, 2005). AtMBD1, 2, 4, 8, 11 cannot specifically bind to 5-mC DNA, while AtMBD4 and AtMBD11 bind to methylated and unmethylated DNAs with or without 5-mC (Ito et al., 2003; Grafi et al., 2007). AtMBD5, 6, 7 show specific binding ability to mCG sites *in vitro* (Zemach and Grafi, 2003). Additionally, AtMBD5 also binds to mCHH sites while AtMBD6 binds to non-specific mCHH and mCHG sites (Ito et al., 2003). The binding activity and specificity have not been established for AtMBD3, 9, 10, 12, and 13 (Ito et al., 2003; Scebba et al., 2003; Grafi et al., 2007). Recently, it was demonstrated that AtMBD6 and AtMBD7 are actually readers for methylated DNA (Wu et al., 2022). AtMBD5 and AtMBD6, which are closely related and may have redundant functions (Berg et al., 2003), are recruited to chromatin by recognition of CG methylation to redundantly repress a subset of genes and transposons (Ichino et al., 2021), or participate in the formation of HDAC complexes to modulate the chromatin structure and gene transcription (Zemach et al., 2005). AtMBD6 also functions in RNA-mediated gene silencing (Parida et al., 2017). AtMBD7 interacts with the histone acetyltransferase Increased DNA Methylation 1 (IDM1) and its partners Increased DNA Methylation 2 (IDM2) and Increased DNA Methylation 3 (IDM3), and participates in DNA demethylation (Lang et al., 2015; Wang et al., 2015). Furthermore, AtMBD7 is also required for the H3K18 and H3K23 acetylation (Li Q. et al., 2015). AtMBD9 recognizes histone acetylation marks by IDM1 and functions in H2A.Z deposition (Nie et al., 2019). Furthermore, the biochemical activities of maize ZmMBD101 and tomato SlMBD5 have also been recently established (Li Y. et al., 2015; Questa et al., 2016).

The functions of *MBD* genes in plant growth, development, and response to abiotic stress have been explored. Mutation in *AtMBD8* or knockdown of *AtMBD11* led to a delay in flowering time, while the *atmbd9* mutant showed a significantly earlier flowering time (Berg et al., 2003; Peng et al., 2006; Stangeland et al., 2009). Overexpression of *Salix viminalis* L. *SvMBD5* led to an early flowering phenotype in transgenic Arabidopsis (Cheng et al., 2020). These observations indicate that the MBD proteins play critical roles in regulation of flowering in plants. The *AtMBD11* knockdown mutant also displayed a variety of phenotypic effects, e.g., aerial rosettes, serrated leaves, abnormal position of flowers, and fertility problems (Berg et al., 2003), while the *atmbd9* mutants produced more shoot branches (Peng et al., 2006). Overexpression of *OsMBD707* leads to larger tiller angles and reduced photoperiod sensitivity in rice (Qu et al., 2021). The *atmbd4* mutant exhibited altered root architecture and up-regulated expression of many phosphate transporters and transcription factors, indicating that *AtMBD4* negatively regulates the phosphate starvation response (Parida et al., 2019). Some of the wheat *TaMBD* genes and most of the petunia *PhMBD* genes were highly induced by abiotic stress and hormones (Hu et al., 2011; Shi et al., 2016, 2022). However, the possible involvement of the *MBD* genes in plant immunity remains elusive.

Watermelon (*Citrullus lanatus* L.) is one of important horticultural crops, providing favorite fresh fruits worldwide. Fusarium wilt, caused by *Fusarium oxysporum* f.sp. *niveum* (*Fon*), and gummy stem blight, caused by *Didymella bryoniae* (*Db*), are two of the most devasting fungal diseases that lead to significant yield losses in watermelon industry (Michielse and Rep, 2009; Keinath, 2011). However, knowledge on the molecular mechanism of resistance in watermelon against *Fon* and *Db* is currently limited, which significantly impedes the breeding for watermelon cultivars with improved resistance against these two fungal diseases. The present study aimed to identify the watermelon *ClMBD* family by characterization and expression analyses and explore the putative mechanism of the *ClMBD* family in disease resistance. The transcript levels of the *ClMBD* genes were changed after treatment with SA and methyl jasmonate (MeJA) and infection by *Fon* and *Db*. Functional analyses revealed that *ClMBD2*, *ClMBD3*, and *ClMBD5* negatively regulate resistance against *Botrytis cinerea* and that *ClMBD1* and *ClMBD2* negatively while *ClMBD5* positively regulate resistance against *Pst* DC3000 in Arabidopsis.

## Materials and Methods

### Plant Materials and Growth Conditions

Watermelon (*Citrullus lanatus*) cv. Zaojia was used for all experiments. *Nicotiana benthamiana* plants expressing a known nucleus-localized marker protein RFP-H2B (Chakrabarty et al., 2007) were used for subcellular localization and bimolecular fluorescence complementation (BiFC) assays. Plants were grown in a soil mix (clay: soil = 3:1) in a growth room under fluorescent light (200 μE m^2^ s^–1^) at 22–24°C with 70% relative humidity (RH) and a 14 h light/10 h dark cycle. Arabidopsis seeds were surface sterilized in 75% ethanol for 5 min and 4% sodium hypochlorite for 10 min, rinsed with sterile water for three times, sowed on 1/2 MS plates and vernalized for 2 days at 4°C. Arabidopsis seedlings were grown on 1/2 MS plates at 22°C with 75% RH with a 16 h light/8 h dark cycle for 7 days and then transplanted to a soil mix (clay: soil = 1:1) in a growth room at 22°C with 75% humidity under a 16 h light/8 h dark cycle for normal growth or under a 8 h light/16 h dark cycle for disease assays.

### Hormone Treatment and Pathogen Inoculation for Gene Expression Analysis in Watermelon

For analysis of tissue-specific expression, leaf, stem and root samples of 4-week-old watermelon plants were collected and stored at −80°C till use. For SA and MeJA treatment, 4-week-old watermelon plants were treated by foliar spraying with 1 mM SA, 100 μM MeJA or an equal volume of solution containing only 0.1% ethanol and 0.02% Tween-20 as controls, and leaf samples were collected at different time points after treatment.

For analysis of gene expression in response to *Fon* infection, pathogen inoculation was performed according to a previously reported method (Song et al., 2015). Briefly, mycelial plugs from 6-day-old culture of *Fon* race 1 strain ZJ1 were transferred into 200 mL mung bean liquid broth (mung bean 20 g/L, boiled for 20 min, pH7.0) and incubated with shaking (250 rpm) at 26°C for 2 days. Spores were collected and spore suspension was adjusted to 1 × 10^7^ spores/mL for inoculation. Three-week-old watermelon plants were uprooted, washed in tap water, the main roots were cut up of one-third, and then dipped for 15 min in *Fon* spore suspension or in mung bean liquid broth as mock-inoculated controls. The inoculated plants were replanted in soil and allowed to grow in the same growth room as described above. Root samples were collected, frozen in liquid nitrogen and stored at −80°C until use.

*Db* strain DBTL4 was grown at 26°C on PNA (potato 200 g/L, NH_4_H_2_PO_4_ 2 g/L, agar 15 g/L, pH7.0) for 6–7 days in dark and then treated with a 12 h UV light/12 h dark cycle for 5 days to induce spore production. After induction, the mycelial plugs were picked into distilled water, spores were collected and the spore suspension was adjusted to 2 × 10^6^ spores/mL. Five-week-old watermelon plants were foliar sprayed with *Db* spore suspension containing 0.05% Tween-20 or with an equal volume of 0.05% Tween-20 solution as mock controls. The inoculated plants were placed in a 22°C chamber with 100% RH for 48 h. Leaf samples were collected, frozen in liquid nitrogen, and stored at −80°C until use.

### Identification of Watermelon *ClMBD* Genes and Proteins

Arabidopsis AtMBD protein sequences were obtained from TAIR^1^ and were used as queries to search for putative MBD genes and proteins in watermelon, melon, cucumber, pumpkin, and zucchini genomes at Cucurbit Genomics Databases.^2^ The obtained nucleotide and protein sequences were examined by domain analysis programs PFAM^3^ (PF01429) and SMART^4^ with the default cutoff parameters. The isoelectric points and molecular weights were predicted on the ExPASy Proteomics Server.^5^ Sequence alignment was carried out by the ClustalX program. Phylogenetic trees were constructed using the neighbor-joining method of the MEGA7 program with the *p*-distance and complete deletion option parameters.

### Synteny Analysis of the *ClMBD* Genes

The reliability of the obtained trees was tested using a bootstrapping method with 1,000 replicates. The MCScanX algorithm with default parameters (Wang et al., 2012) was used to scan orthologous regions containing the watermelon *ClMBD* genes. The corresponding plot was created by Dual Synteny Plot for MCscanX in TBtools software (Chen et al., 2020). The chromosomal localization of *ClMBDs* in the *C. lanatus* genome was obtained by TBtools software (Chen et al., 2020) according to the annotation data of the *C. lanatus* genome. The genomic and annotation data of melon, cucumber, zucchini, and pumpkin were downloaded from the Cucurbit Genomics Database (see text footnote 2), and those of Arabidopsis were downloaded from TAIR (see text footnote 1). The synteny relationship of the orthologous *MBD* genes obtained between watermelon and other selected species was visualized by the Advance Circos package of TBtools (Chen et al., 2020). DnaSP software was used to calculate the non-synonymous (Ka)/synonymous (Ks) values of the duplicated *ClMBD* gene pairs (Librado and Rozas, 2009).

### Cloning of the *ClMBD* Genes

Total RNA was extracted using RNA Isolater reagent (Vazyme, Nanjing, China) according to the manufacturer’s instructions. RNA was treated with RNase-free DNase and then reverse-transcribed into cDNA using the HiScript QRT SuperMix kit (Vazyme, Nanjing, China). The obtained cDNAs were used for cloning, semi-RT-PCR and qRT-PCR. The coding sequences (CDs) of *ClMBDs* were amplified using gene-specific primers (Supplementary Table 1) and cloned into pCAMBIA1300s vector, yielding pCAMBIA1300s-ClMBDs-GFP. After confirmation by sequencing, these pCAMBIA1300s-ClMBDs-GFP plasmids were used as templates to amplify the target genes for further experiments.

### Subcellular Localization Assays

The recombinant pCAMBIA1300s-ClMBDs-GFP plasmids were transformed into *Agrobacterium tumefaciens* strain GV3101. Agrobacteria carrying pCAMBIA1300s-ClMBDs-GFP or pCAMBIA1300s-GFP were separately infiltrated into leaves of *Nicotiana benthamiana* plants expressing a known nucleus-localized marker protein RFP-H2B (Chakrabarty et al., 2007). At 48 h after agroinfiltration, GFP fluorescence signals were excited at 488 nm and detected under a Zeiss LSM780 confocal laser scanning microscope (Zeiss, Oberkochen, Germany) using a 500–530 nm emission filter.

### Yeast Two-Hybrid Assays

Putative interactions between ClMBDs and ClIDM2 or ClIDM3 were examined using the yeast two-hybrid (Y2H) System according to the manufacturer’s instructions (Clontech, Mountain View, CA, United States). The CDs of *ClMBDs* were amplified using gene-specific primers (Supplementary Table 1) from pCAMBIA1300s-ClMBDs-GFP and cloned into pGBKT7 vector, yielding pGBKT7-ClMBDs. ClIDM2 and ClIDM3 were obtained by homologous searching using Arabidopsis AtIDM2 and AtIDM3 as queries and the CDs of ClIDM2 and ClIDM3 were amplified with gene-specific primers (Supplementary Table 1) and cloned into pGADT7 vector, generating pGADT7-ClIDM2 and pGADT7-ClIDM3. The resultant pGBKT7-ClMBD plasmids were transformed with or without pGADT7-ClIDM2 or pGADT7-ClIDM3 into yeast strain Y2HGold by the LiAc/SS carrier DNA/PEG method and confirmed by colony PCR. The transformed yeasts were cultivated on DDO (SD/-Leu/-Trp) medium (Clontech, Mountain View, CA, United States) at 30°C for 3 days, followed by screening on QDO medium containing 40 μg/mL X-α-Gal (Clontech, Mountain View, CA, United States) and 125 ng/mL Aureobasidin A (Clontech, Mountain View, CA, United States). Interactions between ClMBDs and ClIDM2/3 were evaluated according to the growth performance of the transformed yeast cells on QDO and the production of blue pigments after the addition of X-α-Gal. Co-transformation of pGBKT7-53 or pGBKT7-Lam and pGADT7-T were used as positive and negative controls, respectively.

### Bimolecular Fluorescence Complementation Assays

The CDs of *ClMBD2* and *ClMBD3* were amplified using gene-specific primers (Supplementary Table 1) and inserted into p2YN vector, yielding p2YN-ClMBD2 and p2YN-ClMBD3. Similarly, the CDs of *ClIDM2* and *ClIDM3* were inserted into p2YC vector, yielding p2YC-ClIDM2 and p2YC-ClIDM3. Agrobacteria harboring different indicated pairs of plasmids were infiltrated into leaves of *N. benthamiana* plants expressing a red nuclear marker protein RFP-H2B (Chakrabarty et al., 2007). At 48 h after agroinfiltration, YFP and RFP signals were detected and photographed under a Zeiss LSM780 confocal laser scanning microscope (Zeiss, Oberkochen, Germany).

### Electrophoretic Mobility Shift Assays

The CDs of the *ClMBD* genes were amplified using gene-specific primers (Supplementary Table 1) and inserted into pGEX-4T-3 vector, generating pGEX-4T-3-GST-ClMBDs, followed by transforming into *Escherichia coli* strain BL21 (DE3), a widely used non-T7 expression strain that is suitable for transformation and protein expression (New England BioLabs, Beverly, MA, United States). To induce the expression of GST-ClMBD proteins, isopropyl-D-thiogalactoside was added to the bacterial cultures to a final concentration of 1 mM and incubated at 18°C for 20 h. The recombinant GST-ClMBD fusion proteins were purified using glutathione resin columns (Genscript, Shanghai, China) according to the manufacturer’s protocol. The following double-stranded DNA probes were synthesized and used in EMSA assay: 5mCG (GCTCGTAGCTAACGAGCTCGACTCGTTGACATAGGCCAT GGCGTAGACTC) (methylated nucleotides underlined) and its complementary strand with m5C at symmetrical positions, 5mCHG (GCTCTGAGCTAACAGGCTCAGC TCTGTGACATAGGCCATGGCTGAGACTC) (methylated nucleotides underlined) and its complementary strand with m5C at symmetrical positions, 5mCHH (GCTCTTAGCTAACA AGCTCAACTCTATGACATAGGCCATGGCTTAGACTC) (methylated nucleotides underlined) and its complementary strand (GAGTCTAAGCCATGGCCTA TGTCATAGAGGTGAGCTTGTTAGCTAAGAGC) (Ito et al., 2003). Equal volumes of single-stranded DNAs were mixed in annealing buffer (10 mM Tris-HCl, 1 mM EDTA, 100 mM NaCl, pH7.5) and incubated at 85°C for 5 min to form double-stranded DNAs. EMSA was performed as previously described (Yuan et al., 2019) using LightShift Chemiluminescent EMSA Kit (Thermo Fisher Scientific, Waltham, MA, United States). In brief, binding reactions (10 μL) contained 1 μL 10 × binding buffer, 2 μg GST-ClMBD protein or GST protein (as a negative control) and 1 μL biotin-labeled 5mCG, 5mCHG, or 5mCHH probe. In the competitive reactions, unlabeled 5mCG probe was added in excess of 500 times. The binding reactions were incubated at 28°C for 20 min and separated on 6% native PAGE gels. After electrophoresis, the gels were transferred onto Amersham Hybond-N^+^ nylon membrane (GE Healthcare, Buckinghamshire, United Kingdom), and signals from the biotin-labeled probes were detected using a Chemiluminescent Biotin-labeled Nucleic Acid Detection Kit (Beyotime Biotechnology, Haimen, China) according to the manufacturer’s recommendations.

### Generation and Characterization of *ClMBDs*-Overexpressing Transgenic Arabidopsis Lines

Arabidopsis transformation was performed using the floral dip method as previously described (Clough and Bent, 1998). In brief, flowers of 5-week-old Arabidopsis plants were dipped in a suspension of agrobacteria carrying pCAMBIA1300s-ClMBD-GFP plasmids for 1 min. The infected plants were placed in dark for 12 h under 100% RH, returned to the growth room with normal conditions and allowed to grow until the silique maturation. T0 seeds were surface sterilized and then plated on 1/2 MS plates containing 50 μg/mL hygromycin. After treatment at 4°C for 2 days, the plates were transferred to 22°C under a 16 h light/8 h dark cycle, seedlings showing hygromycin resistance, regarded as positive transgenic plants, were transferred in the mixed nutrient soil and allowed for growth for 5–6 weeks to collect seeds. Putative single-copy transgenic lines and homozygous lines were obtained by screening for a 3:1 segregation ratio of hygromycin-resistant (Hgr^R^) character and 100% Hgr^R^ phenotype in T2 and T3 generations on 1/2 MS medium supplemented with 50 μg/mL hygromycin, respectively. The transcript levels of the *ClMBD* genes in the transgenic Arabidopsis lines were analyzed by semi-PCR and qRT-PCR. Two homozygous transgenic Arabidopsis lines with single-copy for each of the *ClMBD* genes (T3 generation) and similar expression levels of the transgenes were chosen for further experiments.

### Disease Assays on Transgenic Arabidopsis Plants and Measurement of *in planta* Pathogen Growth

Disease assays with *B. cinerea* were performed as previously described (Wang et al., 2009). Briefly, spores were collected from 8∼10-day-old culture of *B. cinerea* strain BO5.10 grown on 2 × V_8_ plates and resuspended in 4% maltose and 1% peptone buffer to a final concentration of 2 × 10^5^ spores/mL. Four-week-old Arabidopsis plants were inoculated by foliar spraying with the spore suspension containing 0.05% Tween-20 or with an equal volume of 0.05% Tween-20 solution as mock controls. The inoculated plants were placed in a 22°C chamber with 100% RH for 48 h, and disease development was continuously observed. Measurement of *in planta* fungal growth was performed by analyzing the transcript level of *B. cinerea BcActin* gene and comparing with the transcript level of an Arabidopsis *Actin* gene as an internal control according to a previously reported protocol (Wang et al., 2009).

Disease assays with *Pst* DC3000 were carried out as previously described (Zhang et al., 2016). *Pst* DC3000 was grown on King’s B (KB) broth and bacteria were collected and re-suspended in 10 mM MgCl_2_ solution to OD_600_ = 0.002. The bacterial inoculation was performed by hand infiltration using 1-mL syringes without needle into rosette leaves of 4-week-old Arabidopsis plants and the inoculated plants were kept in sealed containers 22°C for 72 h. For quantification of *in planta* bacterial growth, leaf discs from inoculated leaves were collected and homogenized in 10 mM MgCl_2_. After a series of gradient dilutions, the homogenate was plated on KB plates supplemented with 25 μg/mL rifampicin and bacterial colonies were counted at 3 days after incubation at 28°C.

### *In situ* Detection of H_2_O_2_ Accumulation

Detection of H_2_O_2_ was performed using the DAB staining method (Thordal-Christensen et al., 1997). Leaf samples were collected from Arabidopsis plants with or without infection of *B. cinerea* at 24 h post inoculation (hpi) and dipped into DAB solution (1 mg/mL) in 10 mM Na_2_HPO_4_ (pH7.0). After incubation for 5 h in dark with shaking (80 rpm) at room temperature, the DAB-treated leaves were transferred into acetic acid/glycerol/ethanol (1:1:1, vol/vol/vol) and boiled for 5 min, followed by several washes with the same solution. The DAB-stained leaves were photographed using a digital camera.

### RNA-Seq Analyses

Leaf samples were collected from 4-week-old Col-0 and *ClMBD2*-OE2 Arabidopsis plants, frozen in liquid nitrogen and stored at −80°C. RNA-seq was performed by BioMarker Technologies (Beijing, China) on Hiseq 2500 platform (Illumina). Raw data were filtered to get clean data, sequence comparison with the GCF_000001735.4_TAIR10.1. FPKM (Fragments Per Kilobase of transcript Per Million Fragments Mapped) was used to analyze the level of gene expression (Florea et al., 2013). The expression changes of differentially expressed genes (DEGs) ≥ 1.5-fold and *P*-value < 0.05. Gene Ontology (GO) enrichment analysis of DEGs was implemented by the GOseq R packages based Wallenius non-central hyper-geometric distribution (Young et al., 2010). KOBAS (Mao et al., 2005) software were used to test the statistical enrichment of differential expression genes in KEGG pathways (Kanehisa et al., 2008).

### Semiquantitative RT-PCR and qRT-PCR Analyses

Extraction and treatment of total RNA were performed as mentioned above. Semiquantitative RT-PCR reactions contained 0.5 μL Phanta Max Super-Fidelity DNA Polymerase (Vazyme, Nanjing, China), 0.5 μL dNTP Mix, 12.5 μL 2 × Phanta Max Buffer, 0.1 μg cDNA, 7.5 pmol of each of gene-specific primers (Supplementary Table 1), and 8.5 μL ddH_2_O in a final volume of 25 μL. Arabidopsis *AtActin* was used as the control. Each qPCR reaction contained 10 μL 2 × AceQ qPCR SYBR Green Master Mix (Vazyme, Nanjing, China), 0.1 mg cDNA and 7.5 pmol of each of gene-specific primers (Supplementary Table 1) in a final volume of 20 mL, and had two independent biological replicates. The qPCR was performed in a CFX96 real-time PCR detection system (Bio-Rad, Hercules, CA, United States). Watermelon *ClGAPDH* or Arabidopsis *AtActin* were used as internal controls to normalize the data. Relative gene expression level was calculated using 2^–△△CT^ method as described.

### Statistical Analysis

All experiments were independently repeated three times and the obtained data were subjected to statistical analysis according to the Student’s *t*-test. The probability values of *p* < 0.05 were considered as significant difference between the treatments and corresponding controls.

## Results

### Identification and Characterization of the Watermelon ClMBD Family

To identify putative *ClMBD* genes in watermelon, BLASTp searches were performed against the watermelon genome database using the Arabidopsis AtMBDs as queries and 10 non-redundant sequences that are putative *ClMBD* genes were identified (Table 1). For convenience, unique identities to each of the identified *ClMBD* genes were assigned as *ClMBD1–10* according to their chromosomal locations (Table 1). The CDs of *ClMBD1–10* were confirmed by cloning of the full-length cDNAs using primers designed according to their predicted cDNA sequences. The sizes of the *ClMBD* open reading frames (ORF) ranged from 798 bp (*ClMBD2*) to 6,636 bp (*ClMBD8*) and the sizes of the encoded proteins varied from 265 amino acids (ClMBD2) to 2,211 amino acids (ClMBD8), with molecular weight of 23.34∼244.99 kDa and *p*I of 4.80∼9.49 (Table 1). Similarly, the MBD families in other cucurbit plants were also characterized and 9, 10, 15, and 16 *MBD* genes in melon, cucumber, zucchini, and pumpkin, respectively, were identified (Supplementary Table 2).

**TABLE 1:** Information on the watermelon *ClMBD* family.

Genes	ID	Chromosome	*P*-value	ORF (bp)	Size (aa)	MW (Da)	*p*I	cDNA
*ClMBD1*	Cla97C01G003060	1	1.4e^–19^	2,529	842	94.21	7.88	Yes
*ClMBD2*	Cla97C03G052410	3	2.8e^–13^	798	265	29.34	9.49	Yes
*ClMBD3*	Cla97C05G089970	5	3.8e^–17^	1,782	593	66.08	7.51	Yes
*ClMBD4*	Cla97C06G120480	6	5.7e^–12^	858	285	32.02	4.97	Yes
*ClMBD5*	Cla97C07G139410	7	3.1e^–9^	2,295	764	83.82	5.09	Yes
*ClMBD6*	Cla97C09G165060	9	1.7e^–12^	1,164	387	43.00	4.92	Yes
*ClMBD7*	Cla97C09G169310	9	3.2e^–10^	2,403	800	88.25	8.24	Yes
*ClMBD8*	Cla97C10G197170	10	5.0e^–6^	6,636	2,211	244.99	5.23	Yes
*ClMBD9*	Cla97C11G209600	11	3.9e^–9^	939	312	35.08	4.80	Yes
*ClMBD10*	Cla97C11G217560	11	6.7e^–11^	1,023	340	38.20	5.57	Yes

### Structure of *ClMBD* Genes and Organization of Conserved Domains in ClMBD Proteins

**FIGURE 1:**
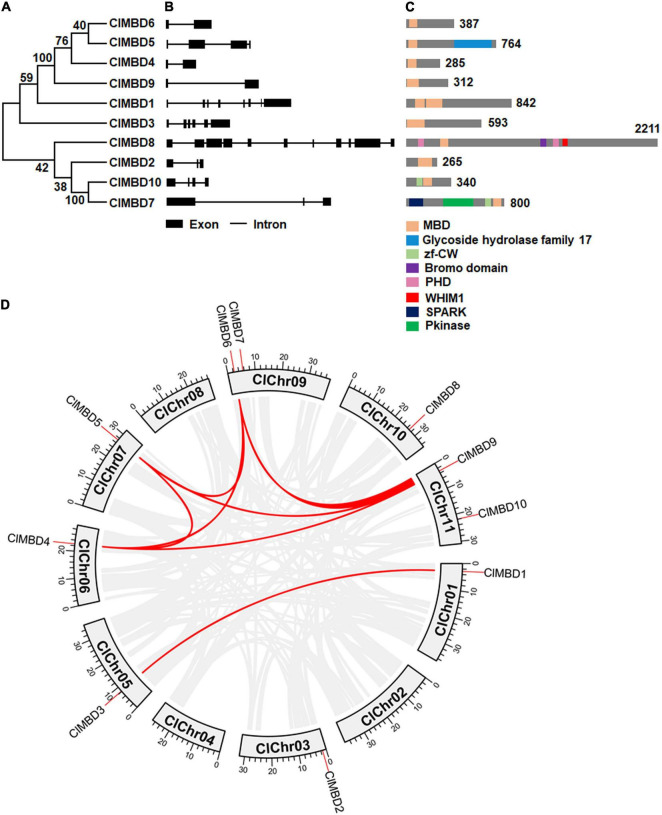
Phylogenetic tree and structure of genes and proteins of the watermelon *ClMBD* family and the evolutionary relationships among the watermelon ClMBD family members. **(A)** Phylogenetic relationships of the watermelon ClMBDs. Clustal X2 program was used for multiple sequence alignments and neighbor joining method of MEGA7 was used for constructing the phylogenic tree. **(B)** Structure of the watermelon *ClMBD* genes. The exon-intron structure of the coding regions of the *ClMBD* genes are shown. Filled boxes indicate exons while lines represent introns. **(C)** Conserved domains and their organization in the watermelon ClMBD proteins. **(D)** Interchromosomal relationships of the watermelon *ClMBD* genes. Gray lines indicate the synteny blocks in the genome, and the red lines indicate tandem duplications of the *ClMBD* gene pairs.

The 10 *ClMBD* genes are unevenly distributed on eight chromosomes in the watermelon genome and chromosomes 2, 4, and 8 do not host any *ClMBD* gene (Table 1 and Supplementary Figure 1). Chromosomes 9 and 11 harbor two *ClMBD* genes while each of the other chromosomes 1, 3, 5, 6, 7, and 10 carry one *ClMBD* gene (Table 1 and Supplementary Figure 1). Phylogenetic tree analysis revealed that the watermelon ClMBD proteins were divided into two clades: Clade I contained six ClMBD proteins (ClMBD1, 3, 4, 5, 6, and 9) while Clade II contained four ClMBD proteins (ClMBD2, 7, 8, and 10) (Figure 1A). Phylogenetic tree analysis of the MBD proteins from cucurbit plants showed that the MBD proteins from watermelon, melon, cucumber, pumpkin, and zucchini have a high level of similarity in the amino acid sequences (Supplementary Figure 2).

The structure of the *ClMBD* genes in the CDs is highly divergent in terms of the exon and intron numbers, with two (*ClMBD9*) to 11 (*ClMBD8*) exons and one (*ClMBD9*) to 10 (*ClMBD8*) introns (Figure 1B). A similar diverse exon-intron structure was also observed in the *MBD* genes in other cucurbit plants such as melon, cucumber, zucchini, and pumpkin (Supplementary Figure 3). The divergent gene structure may imply that the *MBD* genes in cucurbit plants possess divergent functions during their evolution.

The ClMBD proteins contain a characteristic conserved MBD domain (Figure 1C), ranging from 60 to 125 aa in size. The MBD domains in ClMBDs show 13∼43% of sequence identity and harbor some conserved amino acids, e.g., 15W/F, 35Y/F, 38P, and 54L/V (Supplementary Figure 4). Notably, ClMBD1 and ClMBD3 have two MBD domains while the other ClMBDs contain a single MBD domain (Figure 1C). Except for ClMBD7 whose MBD domain locates at the C-terminal, the MBD domains are generally located as the N-terminals in the ClMBD proteins (Figure 1C). ClMBD1, 2, 3, 4, 6, and 9 harbor the sole conserved MBD domains, while ClMBD5, 7, 8, and 10 contain other conserved domains in addition to the MBD domain (Figure 1C). For example, ClMBD5 has a Glycoside Hydrolase Family 17 domain; ClMBD10 has a zf-CW domain; ClMBD7 has a SPARK domain, a PKINase domain, a zf-CW domain; ClMBD8 has a Bromo Domain, a WHIM1 domains and 2 PHD domains (Figure 1C). Similar features in the presence of conserved amino acids in MBD domains (Supplementary Figure 4) and of the additional conserved domains in MBD proteins from melon, cucumber, zucchini, and pumpkin (Supplementary Figure 5) were also detected. The divergence of conserved domains between watermelon and other cucurbit plants may result in the diversity of functions and complexity of the biochemical and molecular mechanisms of the MBD proteins in plants.

### Evolution and Interspecific Synteny of the Watermelon *ClMBD* Family

**FIGURE 2:**
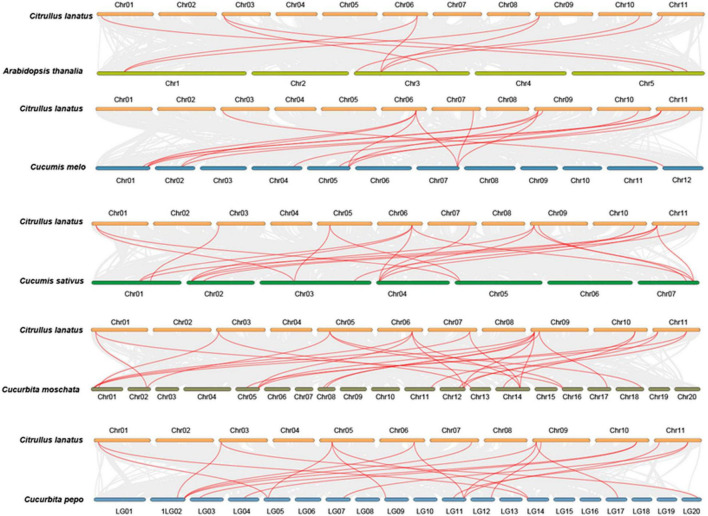
Syntenic relationships of the watermelon *ClMBD* genes with other *MBD* genes in representative plant species. Gray lines in the background indicate the collinear blocks within the watermelon and other plant genomes, while the red lines highlight the syntenic *MBD* gene pairs between watermelon and other plant species.

Gene duplication events in *ClMBDs* in the watermelon genome were detected and seven gene pairs, *ClMBD1/3*, *ClMBD4/5, ClMBD4/6*, *ClMBD4/9*, *ClMBD5/6*, *ClMBD5/9*, and *ClMBD6/9*, were localized in duplicated genomic regions (Figure 1D), implying the occurrence of gene duplication during the evolution of the *ClMBD* gene family in watermelon. The Ka/Ks ratios of *ClMBD1/3*, *ClMBD4/6*, *ClMBD4/9*, *ClMBD5/6*, *ClMBD5/9*, and *ClMBD6/9* were < 1 (Supplementary Table 3), indicating that these gene pairs evolved through purifying selection. Interspecific collinearity analyses identified 9, 15, 20, 19, and 26 collinear gene pairs between watermelon and other tested plant species Arabidopsis, melon, cucumber, zucchini, and pumpkin, respectively (Figure 2 and Supplementary Table 4). Some *ClMBD* genes, e.g., *ClMBD4* and *ClMBD6*, were found to be associated with at least 15 collinear gene pairs identified between watermelon and other tested plant species (Supplementary Table 4), indicating that *ClMBD4* and *ClMBD6* may play essential roles during evolution of the *ClMBD* genes. *ClMBD2*, *4*, *6*, *8*, and *9* showed syntenic relationships with corresponding *MBD* genes in Arabidopsis and other cucurbit crops (Figure 2 and Supplementary Table 4), implying that these pairs of collinear genes may already exist before the ancestral divergence. Particularly, a total of 19,252 collinear gene pairs were identified between watermelon and melon, and 8 watermelon *ClMBD* genes on 6 chromosomes (Chr03, Chr06, Chr07, Chr09, Chr10, and Chr11) and 8 melon *CmMBD* genes on 6 chromosomes (Chr01, Chr02, Chr04, Chr05, Chr07, and Chr12) constituted 15 collinear gene pairs (Figure 2 and Supplementary Table 4). Four colinear gene pairs of watermelon *ClMBDs* distributed on each of Chr06, Chro9, and Chr11, while one colinear gene pair existed on each of Chr03, Chr07, and Chr10 (Figure 2 and Supplementary Table 4). These genes may originate from the same ancestors. Overall, there are more collinear gene pairs between watermelon and other cucurbit plants, indicating that these species were associated with the phylogenetic relationship and that the *ClMBD* gene family may be considered as marker genes in plant evolutionary.

### ClMBDs Are Nucleus-Localized Proteins

**FIGURE 3:**
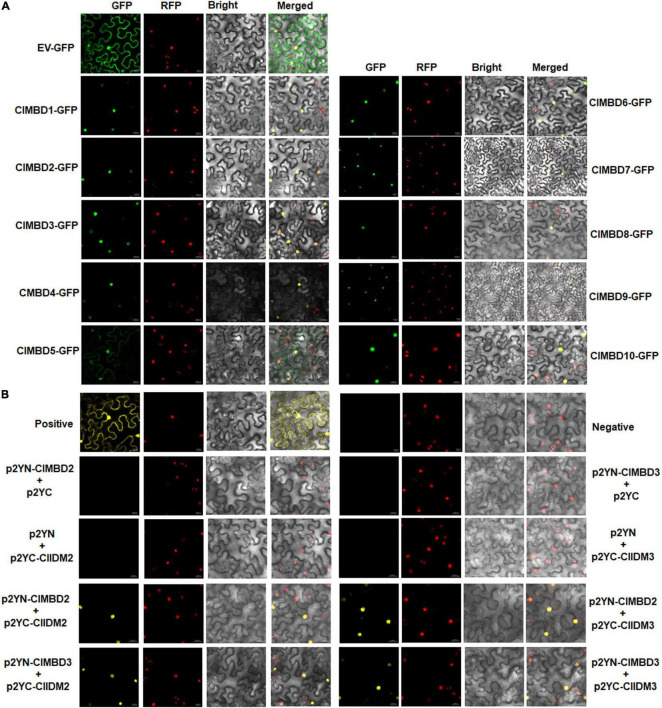
Subcellular localization of the ClMBD proteins and the interactions between ClMBD2/3 and ClIDM2/3. **(A)** ClMBDs are nucleus-localized proteins. Agrobacteria carrying pCAMBIA1300-ClMBDs-GFP or pCAMBIA1300-GFP were infiltrated into leaves of *N. benthamiana* plants expressing a known nucleus-localized marker protein RFP-H2B. At 48 h after agroinfiltration, GFP signals were visualized under a confocal laser scanning microscope in dark field for green fluorescence (*left*), red fluorescence (*middle left*), white field for cell morphology (*middle right*) and in combination (*right*), respectively. **(B)** Interactions of ClMBD2 and ClMBD3 with ClIDM2 and ClIDM3. Agrobacteria carrying indicated pairs of p2YC and p2YN plasmids were infiltrated into leaves of *N. benthamiana* plants expressing a red nuclear marker RFP-H2B protein, and YFP and RFP signals were observed at 48 h after infiltration. Images were taken in dark field for green fluorescence (*left*) and red fluorescence (*middle right*), white field for cell morphology (*middle left*) and in combination (*right*), respectively. Experiments in **(A,B)** were repeated for three times with similar results.

To explore the subcellular localization of the ClMBD proteins, agrobacteria carrying ClMBD1–10-GFP or GFP was infiltrated into leaves of *N. benthamiana* plants expressing a red nuclear marker RFP-H2B protein (Chakrabarty et al., 2007). The ClMBD1–10-GFP protein was solely localized to the nucleus, which was co-localized with the known nucleus marker RFP-H2B protein (Figure 3A). By contrast, GFP alone distributed ubiquitously throughout the cell without specific compartmental localization (Figure 3A). These results indicate that ClMBD1-ClMBD10 are nucleus-localized proteins.

### Interactions Between ClMBDs and ClIDM2/3

The Arabidopsis AtMBD7 interacts with AtIDM2 and AtIDM3 to activate ROS1 to function in demethylation (Lang et al., 2015). To examine whether the ClMBD proteins have similar properties, the interactions of ClMBDs with ClIDM2 and ClIDM3 were examined. In Y2H assays, ClMBD2 and ClMBD3 interacted with ClIDM2 and ClIDM3, but the remaining ClMBDs did not (Supplementary Figure 6). Due to self-activation of ClMBD2 and ClIDM3 in Y2H, the interactions of ClMBD2 and ClMBD3 with ClIDM2 and ClIDM3 were further confirmed using the BiFC assays. YFP signal was not detected in *N. benthamiana* leaves co-infiltrated with agrobacteria harboring p2YN-ClMBD2 + p2YC, p2YN + p2YC-ClIDM2, p2YN-ClMBD3 + p2YC, or p2YN + p2YC-ClIDM3; by contrast, like that in the positive control, significant YFP fluorescence was clearly observed in leaves co-infiltrated with agrobacteria carrying p2YN-ClMBD2 + p2YC-ClIDM2, p2YN-ClMBD3 + p2YC-ClIDM2, p2YN-ClMBD2 + p2YC-ClIDM3, or p2YN-ClMBD3 + p2YC-ClIDM3 (Figure 3B). These results confirmed the interactions of ClMBD2 and ClMBD3 with ClIDM2 and ClIDM3.

### ClMBD2 Specifically Binds to Methylated CG DNA

**FIGURE 4:**
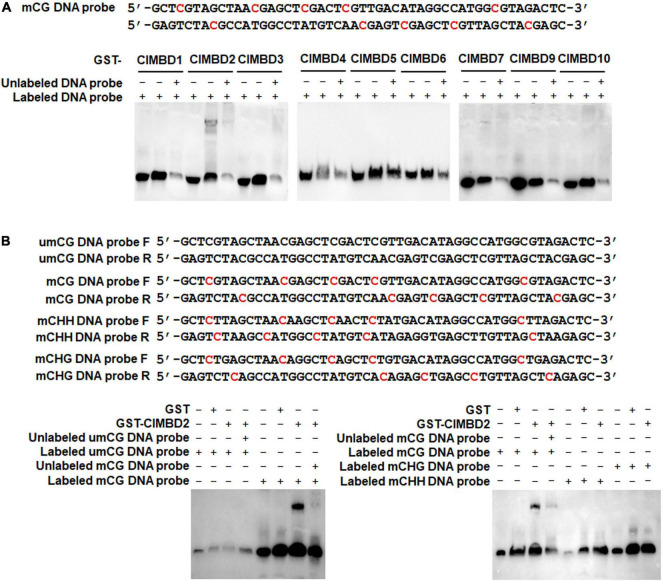
Binding activity of ClMBD2 to methylated CG DNA. **(A)** Binding of ClMBD proteins to the methylated CG DNA. Biotin-labeled mCG DNA (for binding assays) or biotin-labeled mCG DNA in combination with excessive unlabeled mCG DNA (for competitive assays) were incubated with GST-ClMBD or GST (as a negative control). **(B)** Binding activity of ClMBD2 to methylated CG DNA, methylated CHG DNA and methylated CHH DNA. Biotin-labeled mCG DNA, biotin-labeled mCHG DNA and biotin-labeled mCHH DNA (for binding assays) or biotin-labeled mCG DNA in combination with excessive unlabeled mCG DNA (for competitive assays) were incubated with GST-ClMBD or GST (as a negative control). The mCG, mCHG, and mCHH DNA sequences are shown and the methylated sites are indicated in red color. Experiments in **(A,B)** were repeated for three times with similar results.

It is well known that MBD proteins have the capability to bind methylated DNA (Ito et al., 2003; Zemach and Grafi, 2003; Grafi et al., 2007). To explore the biochemical activity of the watermelon ClMBDs, recombinant GST-tagged ClMBD1–7, 9, 10 proteins were purified (Supplementary Figure 7) and their binding activity to methylated CG DNA was examined by EMSA. Two complementary single-stranded DNA probes with 5 methylated CG sites (5mCG) were synthesized and the double-stranded 5mCG DNA was generated (Figure 4A). In repeated EMSA, only ClMBD2 bound to labeled double-stranded 5mCG DNA, and the binding of ClMBD2 to labeled double-stranded 5mCG DNAs was specific as this binding was completely suppressed by the excessive unlabeled double-stranded 5mCG DNA in the competition binding assay (Figure 4A). The remaining ClMBDs did not show binding activity to the labeled double-stranded 5mCG DNA (Figure 4A). However, ClMBD2 did not bind to DNA harboring mCHG or mCHH sites (Figure 4B). These results indicate that ClMBD2 specifically binds to mCG DNA, but not to mCHG DNA or mCHH DNA.

### *ClMBDs* Have Similar Expression Patterns in Root, Stem, and Leaf Tissues

The expression patterns of the *ClMBD* gene in root, stem and leaf tissues of 4-week-old watermelon plants were analyzed and the qRT-PCR results showed that the *ClMBD* genes have similar expression patterns: highest expression in leaves, moderate in stems, and lowest in root (Supplementary Figure 8).

### *ClMBDs* Are Responsive to Defense Hormones Salicylic Acid and Methyl Jasmonate

**FIGURE 5:**
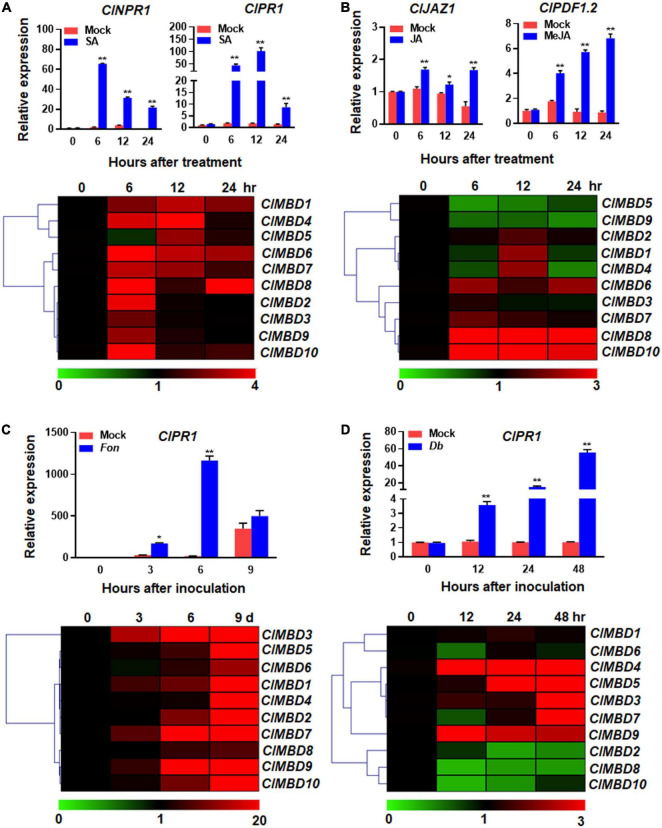
Expression changes of the watermelon *ClMBD* genes in response to defense hormones and fungal pathogens. **(A,B)** Expression changes of the *ClMBD* genes to SA **(A)** or MeJA **(B)**. Four-week-old watermelon plants were treated by foliar spraying with 1 mM SA, 100 μM MeJA or similar volume of solution (as mock controls) and leaf samples were collected at indicated time points after treatment. **(C)** Expression changes of the *ClMBD* genes to *Fusarium oxysporum* f.sp. *niveum*. Three-week-old plants were inoculated by dipping the roots in spore suspension (1 × 10^7^ spores/mL) of *F. oxysporum* f.sp. *niveum* or in mung bean liquid broth as mock-inoculated controls, and root samples were collected at indicated time points after inoculation. **(D)** Expression changes of the *ClMBD* genes to *Didymella bryoniae*. Five-week-old watermelon plants were inoculated by foliar spraying with *D. bryoniae* spore suspension (2 × 10^6^ spores/mL) or similar volume of solution as mock controls, and leaf samples were collected at indicated time points after inoculation. qRT-PCR was performed using the watermelon *ClGAPDH* gene as an internal control. Relative expression was calculated using the 2^–ΔΔCT^ method. Experiments were repeated for three times and the data presented are the means ± SE from three independent experiments. * or ** above the columns indicate significant difference at *p* < 0.05 or *p* < 0.01 levels (Student’s *t*-test), respectively, between treatment/inoculation and mock controls at the same time point.

To explore the possible involvement of the *ClMBD* genes in disease resistance, expression changes of the *ClMBD* genes were analyzed in watermelon plants after treatment with SA and MeJA. After foliar spraying with 1 mM SA, the expression of *ClNPR1* and *ClPR1*, the marker genes of SA signaling pathway, was significantly up-regulated. Particularly, the expression of *ClPR1* significantly up-regulated at 6 h after treatment and peaked at 12 h, showing a > 59-fold increase, as compared with that in mock control (Figure 5A). After SA treatment, the expression of most of the *ClMBD* genes were up-regulated with distinct patterns: *ClMBD2*, *3*, *6*, *7*, *8*, *9*, and *10* were up-regulated at 6 h; *ClMBD1*, *4*, *5*, *7*, and *10* were up-regulated at 12 h; while *ClMBD1*, *6*, and *8* were markedly up-regulated at 24 h, as compared with those in the mock controls (Figure 5A). Notably, *ClMBD2*, *3*, *9*, and *10* showed similar expression patterns after SA treatment, implying that these *ClMBD* genes may have similar functions. These data indicate that the *ClMBD* genes can respond to SA and thus may be involved in disease resistance in watermelon.

After foliar spraying with 100 μM MeJA, the expression of *ClJAZ1* and *ClPDF1.2*, the marker genes of the JA signaling pathway (Yang et al., 2019), was significantly up-regulated and peaked at 24 h (Figure 5B). After MeJA treatment, *ClMBD2*, *6*, *7*, *8*, and *10* were highly up-regulated, while *ClMBD5* and *ClMBD9* were significantly down-regulated, as compared with those in the mock controls (Figure 5B). *ClMBD4* was up-regulated at 12 h but down-regulated at 24 h, as compared with those in the mock controls (Figure 5B). Notably, *ClMBD8*/*ClMBD10* and *ClMBD1*/*ClMBD4* exhibited similar expression patterns in response to exogenous MeJA, indicating these two pairs of the *ClMBD* genes may have similar biological functions. These data indicate that the *ClMBD* genes differentially respond to MeJA and thus may play different roles in disease resistance in watermelon.

### *ClMBDs* Differentially Respond to Fungal Pathogens

To explore the possible involvement of *ClMBDs* in watermelon disease resistance, the expression changes of the *ClMBD* genes in watermelon plants after infection with different fungal pathogens were analyzed. *Fon* is the most important soilborne vascular pathogen that causes Fusarium wilt, one of the most devastating fungal diseases in watermelon (Michielse and Rep, 2009). *Fon* infects watermelon plants through root system and proliferation within the xylem vessels (Michielse and Rep, 2009). Therefore, the expression changes of the *ClMBD* genes in root tissues of watermelon plants after *Fon* infection were analyzed. The qRT-PCR results showed that the expression level of *ClPR1* started to increase at 3 days post inoculation (dpi), peaked at 6 days, leading to 65.5-fold higher over that in mock-inoculated plants, and then decreased (Figure 5C). The expression of *ClMBD1* and *ClMBD10* in root tissues of the *Fon*-infected watermelon plants were up-regulated, as compared with those in the mock-inoculated plants, at 3 dpi (Figure 5C). As compared with those in the mock-inoculated plants, the expression of *ClMBD2*, *6*, and *9* was up-regulated at 6 dpi, while the expression of the *ClMBD* genes, except for *ClMBD3*, *5*, and *10*, was up-regulated at 9 dpi (Figure 5C). Overall, the expression changes of the *ClMBD* genes exhibited similar significant up-regulation patterns in root tissue at 3, 6, or 9 dpi; for example, the expression level of *ClMBD7* in root tissue was markedly up-regulated with a > 639-fold increase than that in mock-inoculated plants at the 9 dpi (Figure 5C). Notably, the expression changes of *ClMBD8*, *9*, and *10*, and of *ClMBD1*, *2*, *4*, *5*, *6*, and *7* showed similar patterns in response to *Fon*, implying that these two groups of the *ClMBD* genes may be involved in resistance with similar functions. The results suggest that the *ClMBD* genes are responsive to *Fon* infection during the relative late stage of the pathogenesis and thus may play roles in the process of regulating watermelon resistance to vascular Fusarium wilt disease.

*Db* is another devasting fungal pathogen that infects leaf and stem tissues and causes gummy stem blight, which is a very common fungal disease on cucurbitaceous crops including watermelon (Keinath, 2011). The responsiveness of the *ClMBD* genes to *Db* infection was also analyzed in leaf tissues of watermelon plants after foliar spraying with a fungal spore suspension. After *Db* inoculation, the expression of *ClPR1* in leaf tissues started to increase at 12 hpi, gradually increased and peaked at 48 h, leading to a 55-fold increase over that in mock-inoculated plants (Figure 5D). The expression of the *ClMBD* genes in leaf tissues exhibited distinct patterns in response to *Db* infection. The expression levels of *ClMBD3*, *4*, *7*, and *9* in *Db*-infected leaves were significantly up-regulated at 12, 24, or 48 hpi, as compared with those in mock-inoculated leaves (Figure 5D). By contrast, the expression levels of *ClMBD2*, *6*, *8*, and *10* in *Db*-infected leaves were highly down-regulated at 12, 24, or 48 hpi, as compared with those in mock-inoculated leaves (Figure 5D). The expression of *ClMBD1* and *ClMBD5* in leaf tissues was not significantly affected by *Db* infection during a 48 h period of the experiments (Figure 5D). Notably, the expression changes of *ClMBD3*, *5*, and *7* exhibited similar patterns in response to *Db*, indicating similar involvement for these three *ClMBD* genes in *Db* resistance. These results indicate that the expression of the *ClMBD* genes in leaf tissues exhibited distinct patterns in response to *Db* infection and therefore may play different roles in the process of regulating watermelon resistance against *Db*.

### Generation and Characterization of *ClMBD*-Overexpressing Arabidopsis Lines

To investigate the functions of the *ClMBD* genes, transgenic Arabidopsis lines with overexpression of an individual *ClMBD* gene were generated. The *ClMBD* genes were transcribed normally in their own transgenic Arabidopsis lines (Supplementary Figures 9A,B). The *ClMBD*-overexpressing Arabidopsis plants showed no significant defect in growth and development, including plant height and size, in comparison to WT plants, when grown in a greenhouse (Supplementary Figure 10).

### *ClMBD2*, *ClMBD3*, and *ClMBD5* Negatively Regulate Arabidopsis Immunity Against *Botrytis cinerea*

**FIGURE 6:**
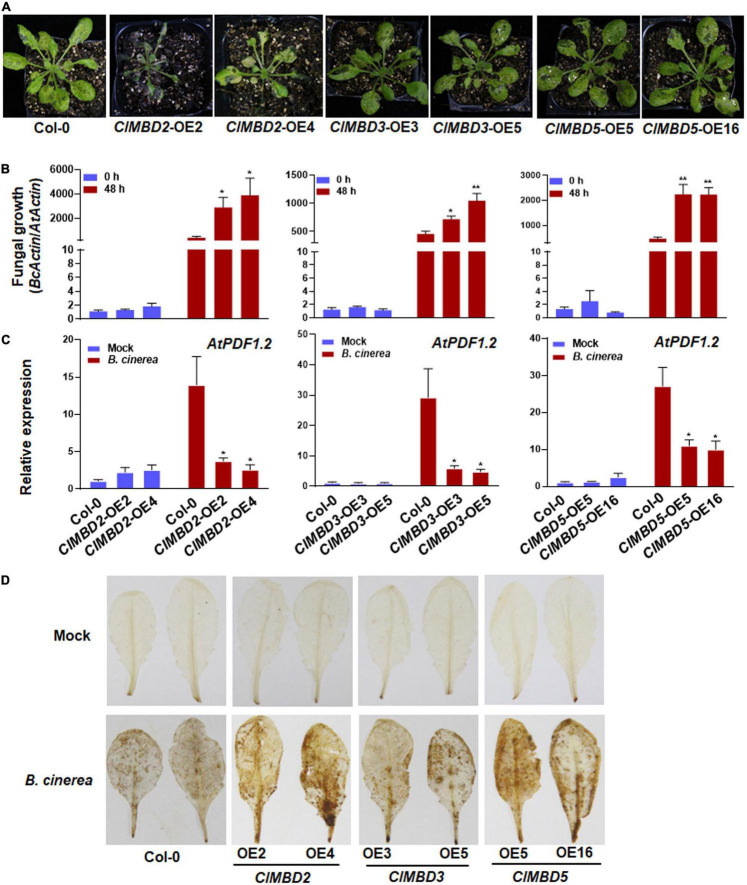
*ClMBD2*, *ClMBD3*, and *ClMBD5* negatively regulate resistance of the transgenic Arabidopsis plants against *Botrytis cinerea*. **(A)** Typical *B. cinerea*-caused disease on WT, *ClMBD2*-OE, *ClMBD3*-OE, and *ClMBD5*-OE plants. Four-week-old plants were inoculated by foliar spraying with *B. cinerea* spore suspension (2 × 10^5^ spores/mL) and photographed at 3 dpi. **(B)** *In planta* fungal growth in inoculated plants. Fungal growth was shown as ratios of the transcript level of *B. cinerea BcActin* to that of the Arabidopsis *AtActin*. **(C)** Relative expression of *AtPDF1.2* in the mock- and *B. cinerea*-inoculated plants. qRT-RCR was performed using *AtActin* as an internal control to analyze the expression level of *AtPDF1.2*. **(D)** Accumulation of H_2_O_2_ in leaves of the in mock- and *B. cinerea*-inoculated plants, as revealed by DAB staining at 24 hpi. Experiments in **(A,D)** were repeated for three times with similar results, and results from one representative experiment are shown. Data presented in **(B,C)** are the means ± SE from three independent experiments and * or ** above the columns indicate significant differences at *p* < 0.05 or *p* < 0.01 levels (Student’s *t*-test), respectively, between the *ClMBD2*/*3*/*5*-OE plants and WT plants at the same time point.

To explore the possible functions of the *ClMBD* genes in plant immunity, disease resistance phenotype of the *ClMBD*-overexpressing Arabidopsis lines and the wild-type (WT) Col-0 plants after infection with *B. cinerea*, a necrotrophic fungus causing grey mold disease, was assessed. In repeated detached leaf punch inoculation assays, *B. cinerea*-caused necrotic lesions on leaves detached from the *ClMBD2*-OE, *ClMBD3*-OE, and *ClMBD5*-OE plants were significantly larger than those on leaves of WT plants, resulting in increases of approximately 88.9, 55.6, and 66.7% in lesion length, respectively, as compared with those on WT leaves (Supplementary Figure 11). By contrast, *B. cinerea*-caused necrotic lesions on leaves detached from the *ClMBD1*-OE, *ClMBD4*-OE, *ClMBD6*-OE *ClMBD7*-OE, *ClMBD8*-OE, *ClMBD9*-OE, and *ClMBD10*-OE plants were comparable to those on leaves of WT plants (Supplementary Figure 11). To confirm these results, the *ClMBD*-overexpressing plants were inoculated by foliar spraying with *B. cinerea* spore suspension and disease severity and fungal growth were compared with those in WT plants. After infection, typical *B. cinerea*-caused disease symptom was seen at 3 dpi. Much severe diseases were observed on leaves of the *ClMBD2*-OE, *ClMBD3*-OE, and *ClMBD5*-OE plants, especially the *B. cinerea*-infected *ClMBD2*-OE plants decayed and died at 3 dpi (Figure 6A). By contrast, disease severity on leaves of the *B. cinerea*-infected *ClMBD1*-OE, *ClMBD4*-OE, *ClMBD6*-OE, *ClMBD7*-OE, *ClMBD8*-OE, *ClMBD9*-OE, and *ClMBD10*-OE plants were similar to that in WT plants (Supplementary Figure 12A). Accordingly, the *ClMBD2*-OE, *ClMBD3*-OE, and *ClMBD5*-OE plants supported more *in planta* fungal growth, leading to increases of 57.5–851.3% over that in WT plants (Figure 6B), while the *ClMBD1*-OE, *ClMBD4*-OE, *ClMBD6*-OE, *ClMBD7*-OE, *ClMBD8*-OE, *ClMBD9*-OE, and *ClMBD10*-OE plants supported similar amounts of *in planta* fungal growth (Supplementary Figure 12B). These data from detached leaf punch inoculation and whole plant inoculation assays indicate that overexpression of *ClMBD2*, *3*, and *5* attenuates the resistance of transgenic Arabidopsis plants against *B. cinerea*, while overexpression of each of the remaining *ClMBD* genes does not affect the resistance against *B. cinerea*.

To get insights in the possible mechanism of the attenuated *B. cinerea* resistance, the expression of a marker defense gene *AtPDF1.2* and accumulation of reactive oxygen species (ROS) were analyzed and compared between the *ClMBD2*-OE, *ClMBD3*-OE, and *ClMBD5*-OE plants and WT plants after infection by *B. cinerea*. In mock-inoculated plants, the expression level of *AtPDF1.2* in the *ClMBD2*-OE, *ClMBD3*-OE, and *ClMBD5*-OE plants was similar to that in WT plants (Figure 6C). At 24 hpi with *B. cinerea*, the expression level of *AtPDF1.2* in the *ClMBD2*-OE, *ClMBD3*-OE, and *ClMBD5*-OE plants and WT plants were markedly up-regulated, as compared with those in the mock-inoculated plants; however, the expression level of *AtPDF1.2* in the *ClMBD2*-OE, *ClMBD3*-OE, and *ClMBD5*-OE plants were significantly suppressed, resulting in a decrease of 73–81, 80–84, and 59–63%, respectively, as compared with that in Col-0 plants (Figure 6C). Similarly, no difference in accumulation of H_2_O_2_, as revealed by *in situ* DAB staining, was observed among the WT, *ClMBD2*-OE, *ClMBD3*-OE, and *ClMBD5*-OE plants without *B. cinerea* challenge (Figure 6D). At 24 hpi with *B. cinerea*, accumulation of H_2_O_2_ increased markedly in *B. cinerea*-infected leaves of the WT, *ClMBD2*-OE, *ClMBD3*-OE, and *ClMBD5*-OE plants, as compared with those in mock-inoculated controls (Figure 6D). However, more staining for H_2_O_2_ in *B. cinerea*-infected leaves of the *ClMBD2*-OE, *ClMBD3*-OE, and *ClMBD5*-OE plants was detected, as compared to that in WT plants (Figure 6D). These data indicate that overexpression of *ClMBD2*, *3*, and *5* in transgenic Arabidopsis plants attenuates the *B. cinerea*-induced expression of defense genes but promotes the *B. cinerea*-induced ROS accumulation.

### *ClMBD1* and *ClMBD2* Negatively but *ClMBD5* Positively Regulate Arabidopsis Immunity Against *Pseudomonas syringae* pv. *tomato* DC3000

**FIGURE 7:**
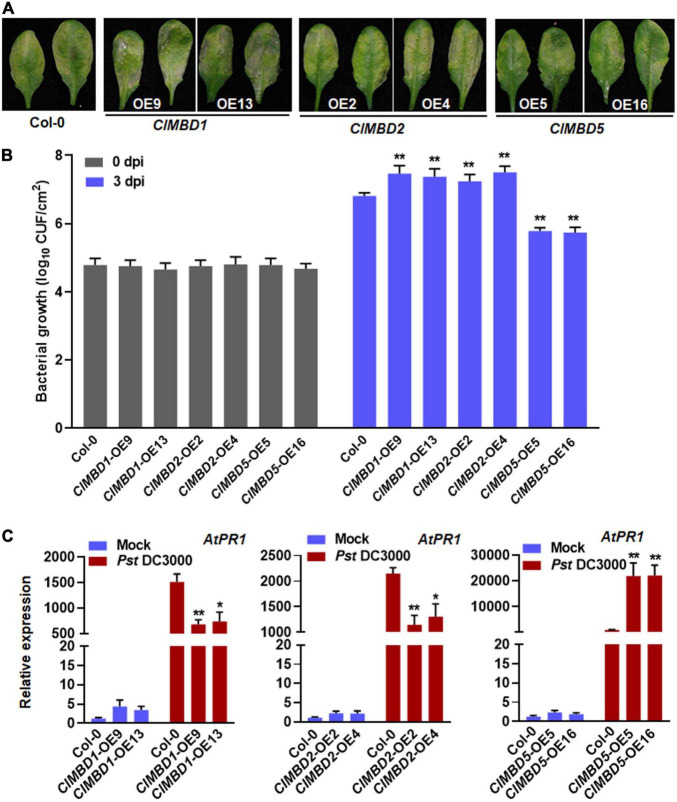
*ClMBD1* and *ClMBD2* negatively but *ClMBD5* positively regulate resistance of the transgenic Arabidopsis plants against *Pseudomonas syringae* pv. *tomato* DC3000. **(A)** Typical *P. syringae* pv. *tomato* DC3000-caused disease on WT, *ClMBD1*-OE, *ClMBD2*-OE, and *ClMBD5*-OE plants. Four-week-old plants were inoculated by injecting with *P. syringae* pv. *tomato* DC3000 bacterial suspension (OD_600_ = 0.0002) and photographed at 72 hpi. **(B)** *In planta* bacterial growth in inoculated leaves. Leaf samples were collected at 0 and 3 dpi and bacterial growth in CFU/cm^2^ leaf area are shown. **(C)** Relative expression of *AtPR1* in the mock- and *P. syringae* pv. *tomato* DC3000-inoculated plants. qRT-RCR was performed using *AtActin* as an internal control to analyze the expression level of *AtPR1* Experiments in **(A,B)** were repeated for three times with similar results, and results from one representative experiment are shown. Data presented in **(C)** are the means ± SE from three independent experiments and * or ** above the columns indicate significant differences at *p* < 0.05 or p < 0.01 levels (Student’s *t*-test), respectively, between the *ClMBD1*/*2*/*5*-OE plants and WT plants at the same time point.

The possible involvement of the *ClMBD* genes in resistance against *Pst* DC3000, a hemibiotrophic pathogen that causes bacterial spot disease, was also investigated. At 3 dpi, typical *Pst* DC3000-provoked symptom with chlorotic lesions was seen in WT plants and the *ClMBD*-overexpressing plants (Figure 7A and Supplementary Figure 13A). The *ClMBD1*-OE and *ClMBD2*-OE plants displayed much severe disease with extensive chlorotic lesion while the *ClMBD5*-OE plants showed less severe disease (Figure 7A). Accordingly, the bacterial growth in the *ClMBD1*-OE and *ClMBD2*-OE plants was 0.43–0.69 order of magnitude higher while the growth in the *ClMBD5*-OE plants was ∼1.0 order of magnitude lower, as compared to that in WT at 3 dpi (Figure 7B). Disease severity and bacterial growth in the *ClMBD3*-OE, *ClMBD4*-OE, *ClMBD6*-OE, *ClMBD7*-OE, *ClMBD8*-OE, *ClMBD9*-OE, and *ClMBD10*-OE plants were indistinguishable to those in WT plants (Supplementary Figure 13B). These data indicate that overexpression of *ClMBD1* and *ClMBD2* leads to attenuated resistance while overexpression of *ClMBD5* results in increased resistance against *Pst* DC3000.

To gain insights in the possible mechanism of the altered *Pst* DC3000 resistance, the expression of a marker defense gene *AtPR1* was analyzed and compared between the *ClMBD1*-OE, *ClMBD2*-OE, and *ClMBD5*-OE plants and WT plants after infection by *Pst* DC3000. In mock-inoculated plants, the expression level of *AtPR1* in the *ClMBD1*-OE, *ClMBD2*-OE, and *ClMBD5*-OE plants was not significantly affected, as compared with that in WT plants (Figure 7C). At 24 hpi with *Pst* DC3000, the expression level of *AtPR1* in the *ClMBD1*-OE, *ClMBD2*-OE, and *ClMBD5*-OE plants and WT plants were markedly up-regulated, as compared with those in the mock-inoculated plants (Figure 7C). However, the *Pst* DC3000-induced expression of *AtPR1* in the *ClMBD1*-OE and *ClMBD2*-OE plants was significantly suppressed, resulting in a decrease of 51–55 and 39–47%, respectively, while the *Pst* DC3000-induced expression of *AtPR1* in the *ClMBD5*-OE plants was markedly increased by ∼21-folds (Figure 7C). These data indicate that overexpression of *ClMBD1* and *ClMBD2* in transgenic Arabidopsis plants attenuates while overexpression of *ClMBD5* strengthens the *Pst* DC3000-induced expression of defense genes.

### Identification of Differentially Expressed Genes in *ClMBD2*-OE Plants

**FIGURE 8:**
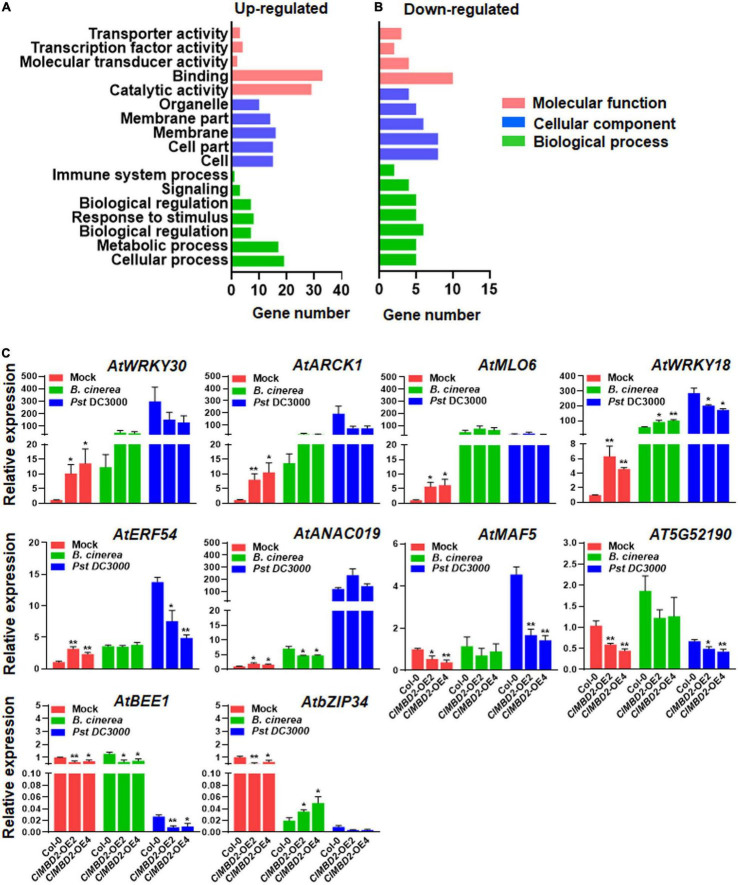
Differentially expressed genes in *ClMBD2*-OE2 plants. **(A,B)** Enriched GO terms of up-regulated **(A)** and down-regulated **(B)** genes in *ClMBD2*-OE2 plants, in comparison to WT plants. **(C)** qRT-PCR validation of expression patterns of selected differentially expressed genes in *ClMBD2*-OE and WT plants with or without infection of *B. cinerea* or *Pst* DC3000 at 24 hpi. Relative expression of the selected genes was analyzed by qRT-RCR using *AtActin* as an internal control. Data presented in **(C)** are the means ± SE from three independent experiments and * or ** above the columns indicate significant differences at *p* < 0.05 or *p* < 0.01 levels (Student’s *t*-test), respectively, between the *ClMBD2*-OE plants and WT plants.

Considering that overexpression of *ClMBD2* led to attenuated resistance against *B. cinerea* and *Pst* DC3000 (Figures 6, 7), transcriptome profiling of the *ClMBD2*-OE2 and WT plants grown under normal growth conditions was performed to gain insights into the possible molecular mechanisms of *ClMBD2* in regulating resistance against the two pathogens. With criteria of expression change > 1.5-folds and *P* < 0.05, a total of 70 genes (52 up-regulated and 18 down-regulated) were found to be differentially expressed genes (DEGs) in the *ClMBD2*-OE2 plants as compared with WT plants (Supplementary Tables 5, 6). The DEGs in the *ClMBD2*-OE2 plants grown under normal condition were categorized into functional groups based on Gene Ontology (GO). DEGs that were up-regulated (Supplementary Table 5) or down-regulated (Supplementary Table 6) in the *ClMBD2*-OE2 plants were clustered into 31 (Supplementary Table 7) and 22 categories (Supplementary Table 8), respectively. Some overrepresented categories include genes involved in DNA binding, molecular transducer activity, and transcriptional factor activity in molecular function category, and response to stimulus, immune system process, signaling, and biological regulation in biological processes category (Figures 8A,B), implying that overexpression of *ClMBD2* in transgenic Arabidopsis plants may affect the immune signaling and response. Among the DEGs (Supplementary Tables 5, 6), some genes have been previously reported to be involved in Arabidopsis immunity, including *AtWRKY18* (Xu et al., 2006), *AtWRKY30* (Zou et al., 2019), *AtWRKY54* (Chen et al., 2021), *AtANAC019* (Zheng et al., 2012), *AtMLO6* (Acevedo-Garcia et al., 2017; Kuhn et al., 2017), and *AtNATA1* (Lou et al., 2016). The expression patterns of 10 selected genes were further validated by qRT-PCR in *ClMBD2-OE* and WT plants before and after the infection of *B. cinerea* and *Pst* DC3000. In the *ClMBD2*-OE plants without pathogen infection, the expression levels of *AtWRKY18*, *AtWRKY30*, *AtANAC019*, *AtARCK1*, *AtMLO6*, and *AtERF54* were significantly up-regulated while the expression levels of *AtMAF5*, *AtBEE1*, *AtbZIP34*, and *AT5G52190* were markedly down-regulated (Figure 8C). After infection of *B. cinerea*, the expression of *AtbZIP34* was down-regulated, while the expression of other genes was up-regulated in *ClMBD2-OE* and WT plants (Figure 8C). The expression levels of *AtWRKY18* and *AtbZIP34* were significantly increased, while the expression levels of *AtANAC019* and *AtBEE1* were significantly suppressed in *ClMBD2*-OE plants after infection of *B. cinerea*, as compared with those in WT plants (Figure 8C). After infection of *Pst DC3000*, the expression of *AtWRKY18*, *AtWRKY30*, *AtANAC019*, *AtARCK1*, *AtERF54*, *AtMLO6*, and *AtMAF5* was up-regulated, but the expression of *AtBEE1*, *AT5G52190*, and *AtbZIP34* was down-regulated in *ClMBD2-OE* and WT plants (Figure 8C). The expression levels of *AtWRKY18*, *AtERF54*, *AtMAF5*, *AT5G52190*, and *AtBEE1* were significantly decreased in *ClMBD2*-OE plants after infection of *Pst* DC3000, as compared with those in the WT plants (Figure 8C). These data consistently conformed the results from RNA-seq analysis and indicate that *ClMBD2* regulates a small set of defense and signaling genes that are involved in Arabidopsis immunity.

## Discussion

It has been documented that the MBD proteins play important roles in plant growth, development, and abiotic stress response; however, the involvement of the MBD proteins in plant immunity has not been established. The present study characterized the MBD families in watermelon and other cucurbit plants, examined the subcellular localization and binding activity of ClMBDs to 5-mC DNAs, analyzed the expression patterns of *ClMBDs* in response to defense hormones and pathogens, and explored the functions of *ClMBDs* in disease resistance. The functional analysis in transgenic Arabidopsis revealed that *ClMBD1*, *2*, *3*, and *5* play roles in immunity against *B. cinerea* and *Pst* DC3000, providing novel insights into the function of the *MBD* genes in plant immunity and a possibility to improve plant disease resistance through genetic manipulation of specific *MBD* genes.

The present study identified 10 watermelon *ClMBD* genes and 9, 10, 15, and 16 *MBD* genes in melon, cucumber, zucchini, and pumpkin, respectively (Table 1 and Supplementary Table 2). The numbers of *ClMBDs* in watermelon and *MBD* genes in other cucurbit plants are comparable to those of Arabidopsis (13), rice (17), maize (14), poplar (14), potato (15), tomato (18), and petunia (11) (Grafi et al., 2007; Parida et al., 2018; Shi et al., 2022). The presence of gene pairs in duplicated genomic regions of the watermelon genome and syntenic collinearity gene pairs between watermelon and other cucurbit plant species (Figures 1, 2 and Supplementary Tables 3, 4) suggests that gene duplication events occurred during the evolution of the *ClMBD* family. In addition to the typical MBD domain, other conserved domains such as zf-CW domain, SPARK domain, PKINase domain, and Bromo domain were also identified in some of the ClMBD proteins (Figure 1). Similar conserved domains are also present in Arabidopsis AtMBDs, tomato SlMBDs, and potato StMBDs (Grafi et al., 2007; Parida et al., 2018). It is thus likely that some watermelon ClMBD proteins may exert their functions in affecting transcription of target genes through different biochemical mechanisms including protein-protein interactions. Subcellular localization observations revealed that the ClMBD proteins were localized in nucleus when transiently expressed in *N. benthamiana* (Figure 3). This is consistent with the previous observations that most of the Arabidopsis AtMBD proteins displayed clear localization within the nucleus in onion cells (Berg et al., 2003; Ito et al., 2003) and that ZmMBD101 localized to nucleoplasmic foci (Questa et al., 2016). Another, ClMBD2 and ClMBD3 interacted with ClIDM2 and ClIDM3, and the interactions occurred in nucleus in BiFC assays (Figure 3). This feature is similar to the interaction of the Arabidopsis AtMBD7 with AtIDM2 and AtIDM3 (Lang et al., 2015), and further confirmed the nuclear localization of the ClMBD2 and ClMBD3. ClMBD2 is phylogenetically related to AtMBD5 and AtMBD6 and also shows an evolutionary syntenic relationship with AtMBD5 (At3G46580) and AtMBD6 (At5G59380) (Supplementary Table 4), implying that ClMBD2 may have a similar biochemical activity to AtMBD5 and AtMBD6. In the present study, ClMBD2 showed the ability to bind to mCG DNA (Figure 4), similar to AtMBD5 and AtMBD6, maize ZmMBD101, and tomato SlMBD5, which have the binding ability to mCG DNA (Ito et al., 2003; Scebba et al., 2003; Grafi et al., 2007; Li Y. et al., 2015; Questa et al., 2016). However, ClMBD2 did not bind to mCHH and mCHG DNA (Figure 4), different from AtMBD5 and AtMBD6, which also have the ability to bind to mCHH DNA (Ito et al., 2003; Scebba et al., 2003; Grafi et al., 2007). Surprisingly, the binding activity of the other ClMBD proteins to mCG DNA was not detected in the present study (Figure 4), implying that the ClMBD proteins may have different biochemical activities in recognizing methylated or unmethylated DNA and thus confer specific biological functions.

It was previously observed that the expression of some tomato *SlMBD*, wheat *TaMBD* and petunia *PhMBD* genes were affected by abscisic acid and abiotic stress, e.g., drought, salt, and cold stress (Li et al., 2008; Hu et al., 2011; Parida et al., 2018; Shi et al., 2022). The expression of most of the watermelon *ClMBD* genes was up-regulated after SA or MeJA treatment, except that *ClMBD5* and *ClMBD9* were down-regulated by MeJA and that the expression of *ClMBD1* and *ClMBD3* was not affected by MeJA (Figure 5). In response to *Fon*, the expression of almost all of the *ClMBD* genes in root tissues was up-regulated (Figure 5). By contrast, the expression of *ClMBD3*, *4*, *7*, and *9* was up-regulated, while the expression of *ClMBD2*, *6*, *8*, and *10* was down-regulated in response to *Db* (Figure 5). Notably, the expression changes of the *ClMBD* genes exhibited differential but inconsistent patterns in leaf and root tissues of watermelon plants in response to treatment of SA and MeJA and to infection of *Fon* and *Db*. However, the expression changes induced by the two defense hormones and the two fungal pathogens imply the involvement of the watermelon *ClMBD* genes in disease resistance, probably through affecting transcription of a set of genes including those involved in defense response.

The responsiveness of the watermelon *ClMBD* genes to exogenous SA and JA, two hormones that mediated defense response against (hemi)biotrophic pathogens such as *Pst* DC3000 and necrotrophic fungi like *B. cinerea* (Glazebrook, 2005; Grant and Jones, 2009) led to evaluate the disease resistance phenotype of the *ClMBD*-OE Arabidopsis lines against *Pst* DC3000 and *B. cinerea*. In the present study, ectopic overexpression of *ClMBD2*, *3*, and *5* in transgenic Arabidopsis led to increased susceptibility to *B. cinerea* (Figure 7 and Supplementary Figure 12), suggesting that *ClMBD2*, *3*, and *5* are negative regulators of defense response against *B. cinerea*. This is further supported by the suppression of pathogen-induced expression of defense gene *AtPDF1.2*, an indicator gene of defense response against necrotrophic fungal pathogens, and overaccumulation of ROS in *ClMBD2*-OE, *ClMBD3*-OE, and *ClMBD5*-OE plants (Figure 6). This is consistent with the general concept that excessive ROS accumulation during early stage often benefits the infection by the necrotrophic fungi like *B. cinerea*, but is different from the phenomenon that early ROS accumulation is critical to the activation of immune response against (hemi)biotrophic pathogens (Mengiste, 2012). On the other hand, overexpression of *ClMBD1* and *ClMBD2* in transgenic Arabidopsis attenuated while overexpression of *ClMBD5* strengthened resistance to *Pst* DC3000 (Figure 7), indicating that *ClMBD1* and *ClMBD2* are negative regulators while *ClMBD5* is a positive regulator of immunity against this bacterial pathogen. This is consistent with the suppression of expression of *AtPR1* in *ClMBD1*-OE and *ClMBD2*-OE plants but elevation of expression of *AtPR1* in *ClMBD5*-OE plants after infection of *Pst* DC3000 (Figure 7). This is also indirectly supported by the down-regulated expression of *ClMBD2* in leaf tissues of watermelon plants after infection of *Db*, a heminecrotrophic fungal pathogen (Figure 5). Notably, overexpression of *ClMBD2* in transgenic Arabidopsis resulted in attenuated immunity against both *B. cinerea* and *Pst* DC3000; however, overexpression of *ClMBD5* led to opposite functions in immunity against these two pathogens, e.g., attenuated immunity against *B. cinerea* but strengthened immunity against *Pst* DC3000 (Figures 6, 7). It is generally accepted that immune response against (hemi)biotrophic pathogens such as *Pst* DC3000 is modulated through the SA signaling while the defense response against necrotrophic pathogens like *B. cinerea* is regulated by the JA/ET signaling (Glazebrook, 2005; Grant and Jones, 2009). Both antagonistic interaction and synergistic cross-talks between the SA and JA/ET signaling pathways occur and allow plants to mount appropriate immune responses against different invading pathogens (Glazebrook, 2005; Koornneef and Pieterse, 2008; Verhage et al., 2010). It is therefore likely that *ClMBD2* and *ClMBD5* function in immunity through regulating different mechanisms.

Transcriptome profiling identified a limited number of DEGs in the *ClMBD2*-OE2 plants grown under normal growth conditions (Supplementary Tables 5, 6). The fact that genes involved in DNA binding, transcriptional factor activity, response to stimulus, and immune system process were overrepresented in DEGs in *ClMBD2*-OE2 plants (Figures 8A,B) further confirms the function of *ClMBD2* in immunity of the transgenic Arabidopsis plants against *B. cinerea* and *Pst* DC3000. Generally, the MBD proteins recognize the methylated CG sites and recruit chromatin remodelers and histone deacetylases to repress transcription of target genes (Lang et al., 2015). Considering the attenuated immunity against *B. cinerea* and *Pst* DC3000 (Figures 6, 7), it is speculated that overexpression of *ClMBD2* should lead to down-regulation of a set of genes that are involved in Arabidopsis immunity. Surprisingly, only 18 genes were identified as down-regulated genes (expression change > 1.5-folds and *P-*value < 0.05) in the *ClMBD2*-OE2 plants (Supplementary Table 6). Among these down-regulated genes, *AtWRKY54* was previously reported to function as a positive regulator of *SARD1* and *CBP60g* expression in immunity against *P. syringae* pv. *maculicola* (Chen et al., 2021; Figure 8). No other gene with known function in Arabidopsis immunity was identified in the down-regulated genes in *ClMBD2*-OE2 plants (Supplementary Table 6). This might be due to the fact that samples from healthy *ClMBD2*-OE2 plants without pathogen infection were used for RNA-seq analysis. Indeed, the expression of defense genes such as *AtPR1* and *AtPDF1.2* was suppressed significantly in the *ClMBD2*-OE plants upon infection with *Pst* DC3000 and *B. cinerea*, respectively (Figures 6, 7). If it is the case that *ClMBD2*, like its closely related *AtMBD5* and *AtMBD6*, acts to repress transcription of target genes, this may imply that the function of *ClMBD2* in suppression of transcription of defense genes in transgenic Arabidopsis plants occurs upon pathogen infection. By contrast, some genes that negatively regulate Arabidopsis immunity were found to be up-regulated in the *ClMBD2*-OE2 plants (Figure 8 and Supplementary Table 5). For example, *AtWRKY18* negatively regulates resistance against *Pst* DC3000 but positively modulates resistance to *B. cinerea* (Xu et al., 2006), *AtANAC019* negatively regulates immune response through repressing *AtICS1* and thus inhibiting SA accumulation (Zheng et al., 2012), *AtMLO6* is a susceptible gene for powdery mildew disease (Acevedo-Garcia et al., 2017; Kuhn et al., 2017), and *AtNATA1* negatively regulates immunity against *Pst* DC3000 by acetylating putrescine and decreasing ROS accumulation (Lou et al., 2016). It seems that overexpression of *ClMBD2* activates an unknown pathway that up-regulates the expression of a subset of genes with negative functions in Arabidopsis immunity.

In summary, the present study characterized the watermelon ClMBD family and the MBD families in other cucurbit plants in terms of gene structures, conserved domain organization, phylogenetic and syntenic relationships, evolution events, subcellular localization, biochemical activity, and expression patterns in response to defense hormones and pathogen infection. The present study also provided the information on the possible involvement of each of the watermelon *ClMBD* genes in disease resistance when they were ectopically expressed in Arabidopsis. Functional analyses in transgenic Arabidopsis revealed that *CMBD2*, *3*, and *5* negatively regulate Arabidopsis resistance against *B. cinerea*, and that *ClMBD1* and *ClMBD2* negatively while *ClMBD5* positively regulate Arabidopsis resistance against *Pst* DC3000. Transcriptome analysis showed that overexpression of *ClMBD2* in transgenic Arabidopsis affected the expression of a small set of genes that are involved in Arabidopsis immunity. Further analyzing the DNA methylation levels and characterizing the genome-wide binding sites in the *ClMBD2*-OE and *ClMBD5*-OE transgenic Arabidopsis plants will definitely provide detailed molecular mechanisms by which *ClMBD2* and *ClMBD5* regulate immunity against *B. cinerea* and *Pst* DC3000. Due to the divergence of gene functions in immunity between Arabidopsis and watermelon, the functional analysis in the present study performed by ectopic overexpression in Arabidopsis may not reflect the intrinsic functions of the *ClMBD* gene in watermelon disease resistance. Therefore, further investigations in watermelon through overexpression and CRISPR/Cas9-based knockout approaches will be critical to elucidate the functions and molecular mechanisms of the *ClMBD* genes, especially the *ClMBD1*, *2*, *3*, and *5* in disease resistance against *Fon*, *Db*, and other pathogens.

## Data Availability Statement

The datasets presented in this study can be found in online repositories. The names of the repository/repositories and accession number(s) can be found below: https://www.ncbi.nlm.nih.gov/, PRJNA803007.

## Author Contributions

FS and DL conceived the project and designed the experiments. JL generated all material used in this study (cloning, vector, transformations, transgenic plants). JL, XL, YW, and XW performed the experiments and collected the data. FS, JL, and DL analyzed the data. FS and JL drafted the manuscript. All authors commented on the manuscript.

## Conflict of Interest

The authors declare that the research was conducted in the absence of any commercial or financial relationships that could be construed as a potential conflict of interest.

## Publisher’s Note

All claims expressed in this article are solely those of the authors and do not necessarily represent those of their affiliated organizations, or those of the publisher, the editors and the reviewers. Any product that may be evaluated in this article, or claim that may be made by its manufacturer, is not guaranteed or endorsed by the publisher.

## Footnotes

1
https://www.arabidopsis.org

2
http://www.icugi.org/

3
http://pfam.xfam.org

4
http://smart.embl-heidelberg.de

5
http://expasy.org/
