# Supplementary material for: Genome-Wide Characterization of the Methyl CpG Binding Domain-Containing Proteins in Watermelon and Functional Analysis of Their Roles in Disease Resistance Through Ectopic Overexpression in Arabidopsis thaliana
Source: Front Plant Sci. 2022 May 9;13:886965. doi: 10.3389/fpls.2022.886965 (PMC9125323; doi:10.3389/fpls.2022.886965)
Supplement: Supplementary file 1 [file Data_Sheet_1.docx]

**Supplementary Materials**

**Genome-Wide Characterization of the Methyl CpG Binding Domain-Containing Proteins in Watermelon and Functional Analysis of Their Roles in Disease Resistance through Ectopic Overexpression in *Arabidopsis thaliana***

Jiayu Liang, Xiaodan Li, Ya Wen, Xinyi Wu, Hui Wang, Dayong Li *, Fengming Song *

**
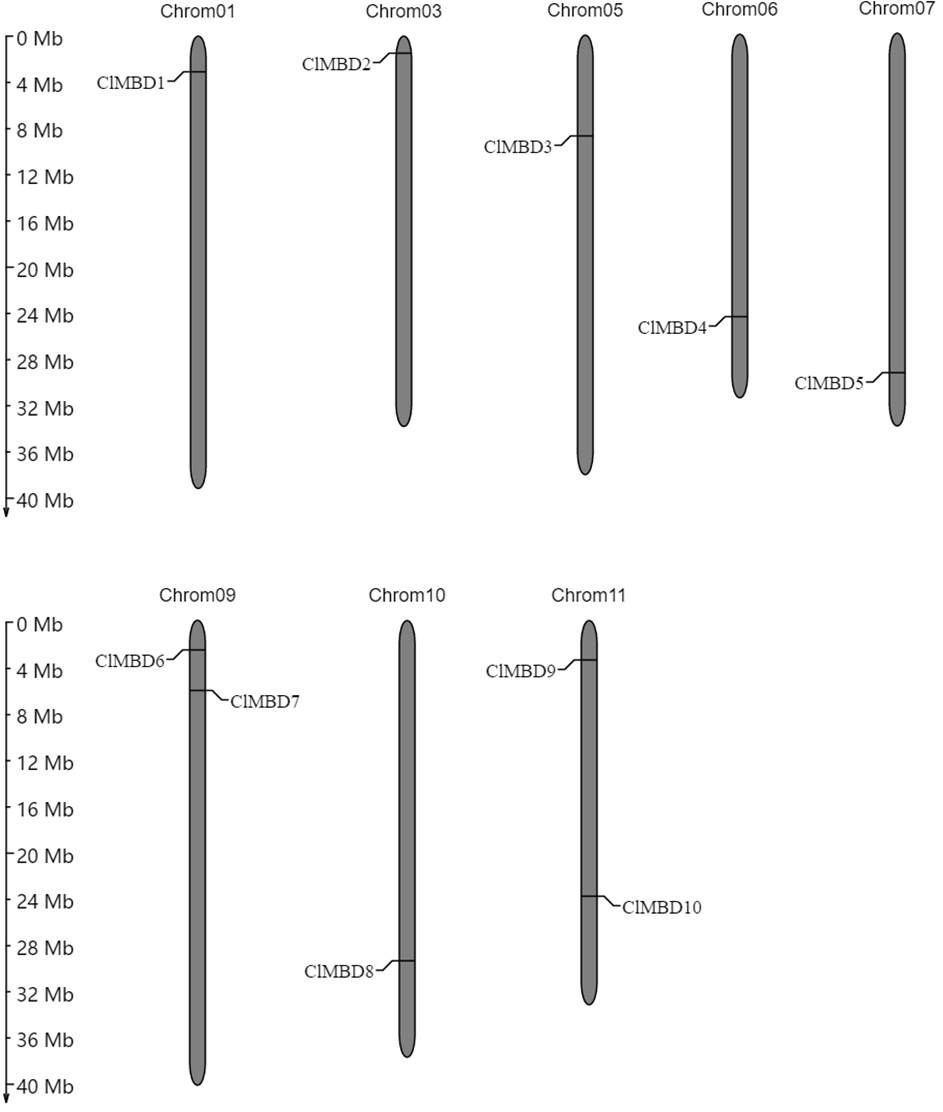
**

**Supplementary Figure S1 |** Distribution of the *ClMBD* genes on the watermelon chromosomes.

**
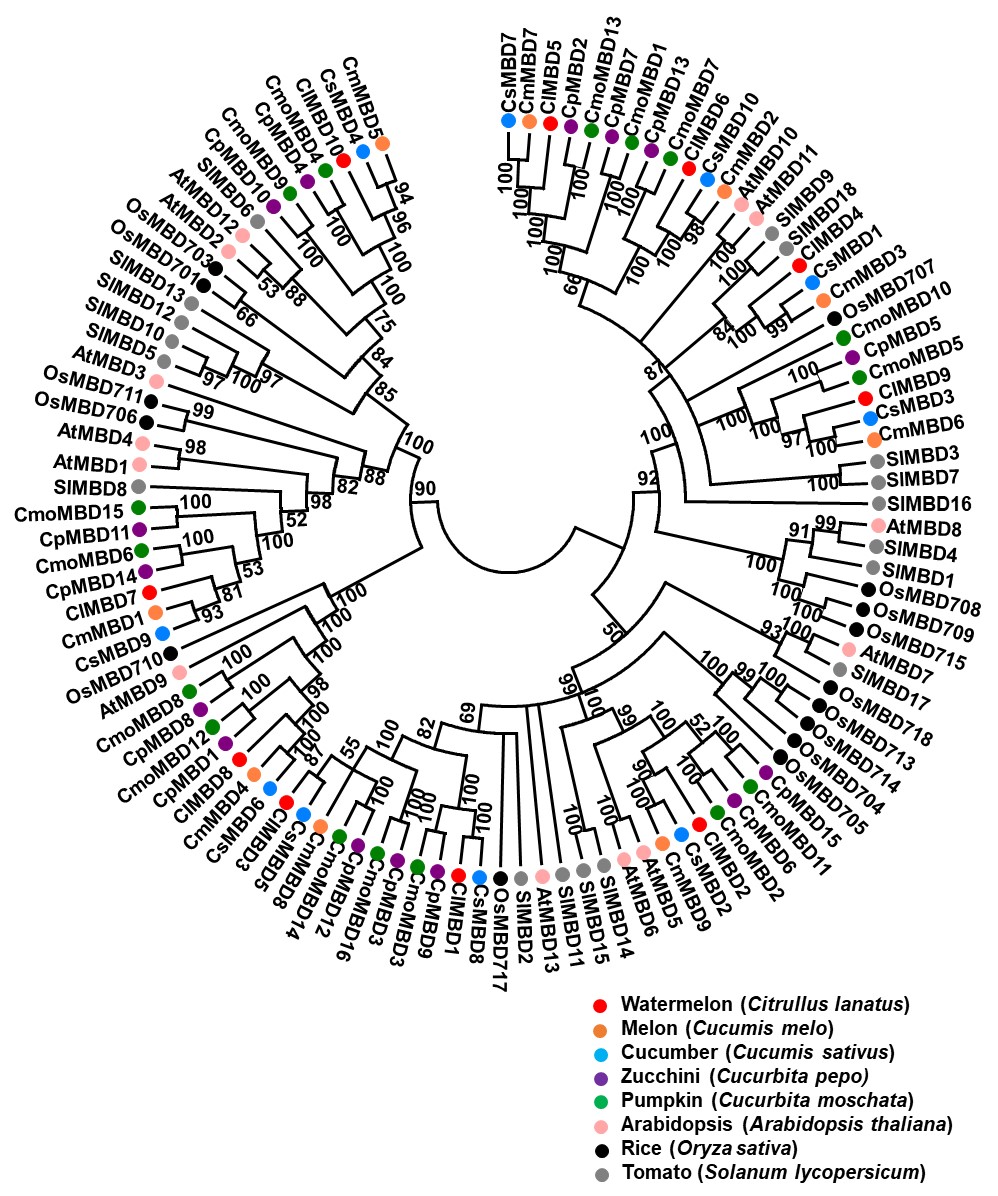
**

**Supplementary Figure S2 |** Phylogenetic tree of the MBD proteins in Arabidopsis, rice, tomato and cucurbit plants. Clustal X2 program was used for multiple sequence alignments and Neighbor-Joining method was used for constructing the phylogenic tree.

**
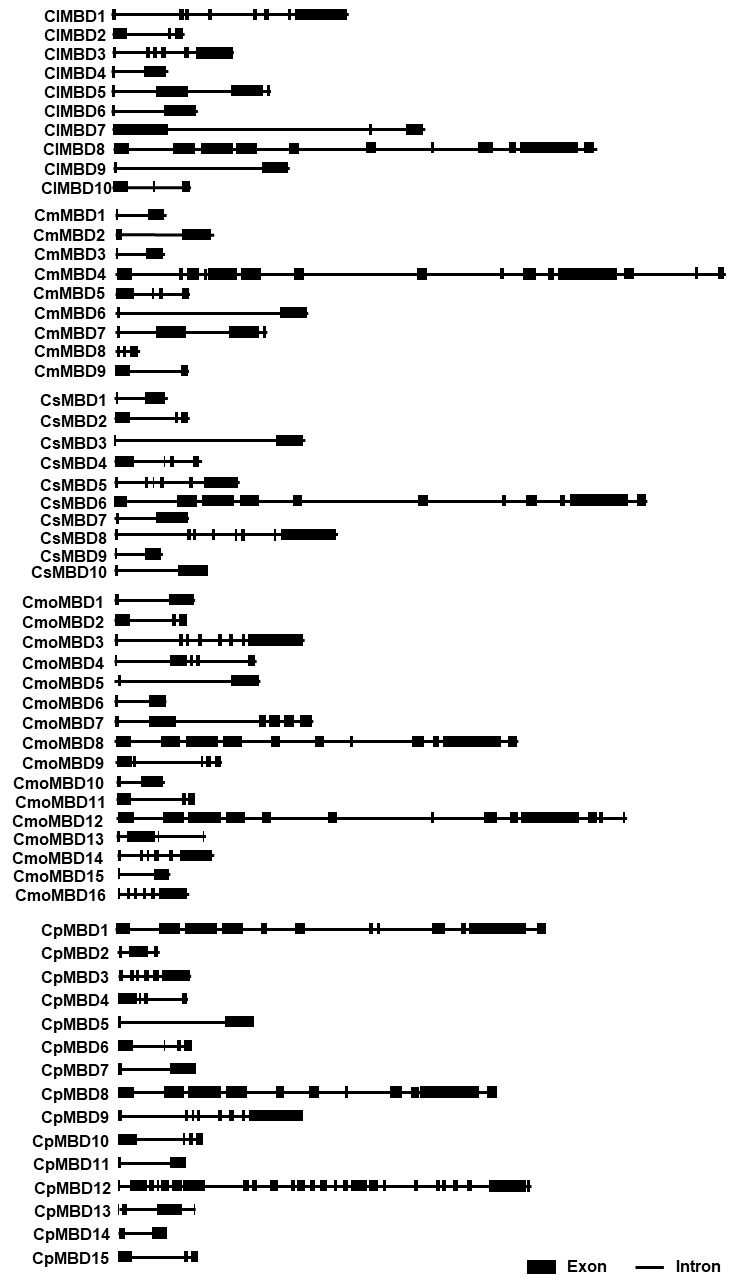
**

**Supplementary Figure S3 |** Exon-intron structures of the MBD genes from watermelon (*Cl*), melon (*Cm*), cucumber (*Cs*), zucchini (*Cp*), and pumpkin (*Cmo*). Filled boxes indicate exons while lines represent introns.

**
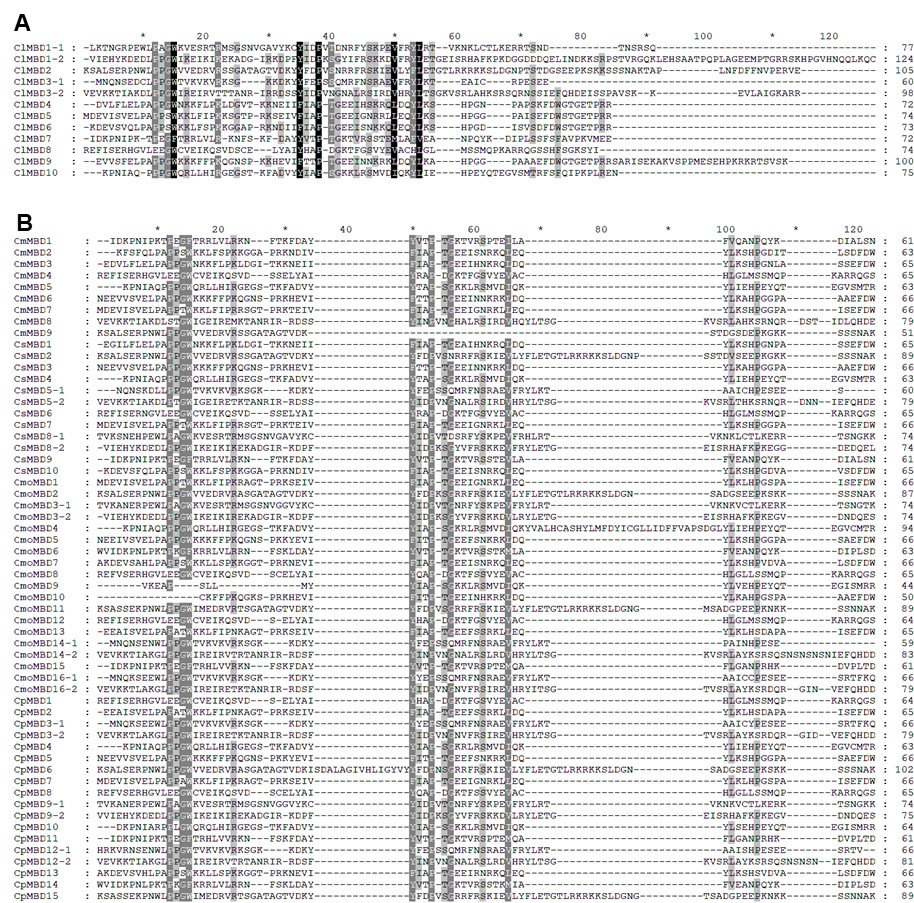
**

**Supplementary Figure S4 |** Sequence alignment of the MBD domains in ClMBD proteins from watermelon (**A**) and MBD proteins from melon (Cm), cucumber (Cs), zucchini (Cp), and pumpkin (Cmo) (**B**).

**
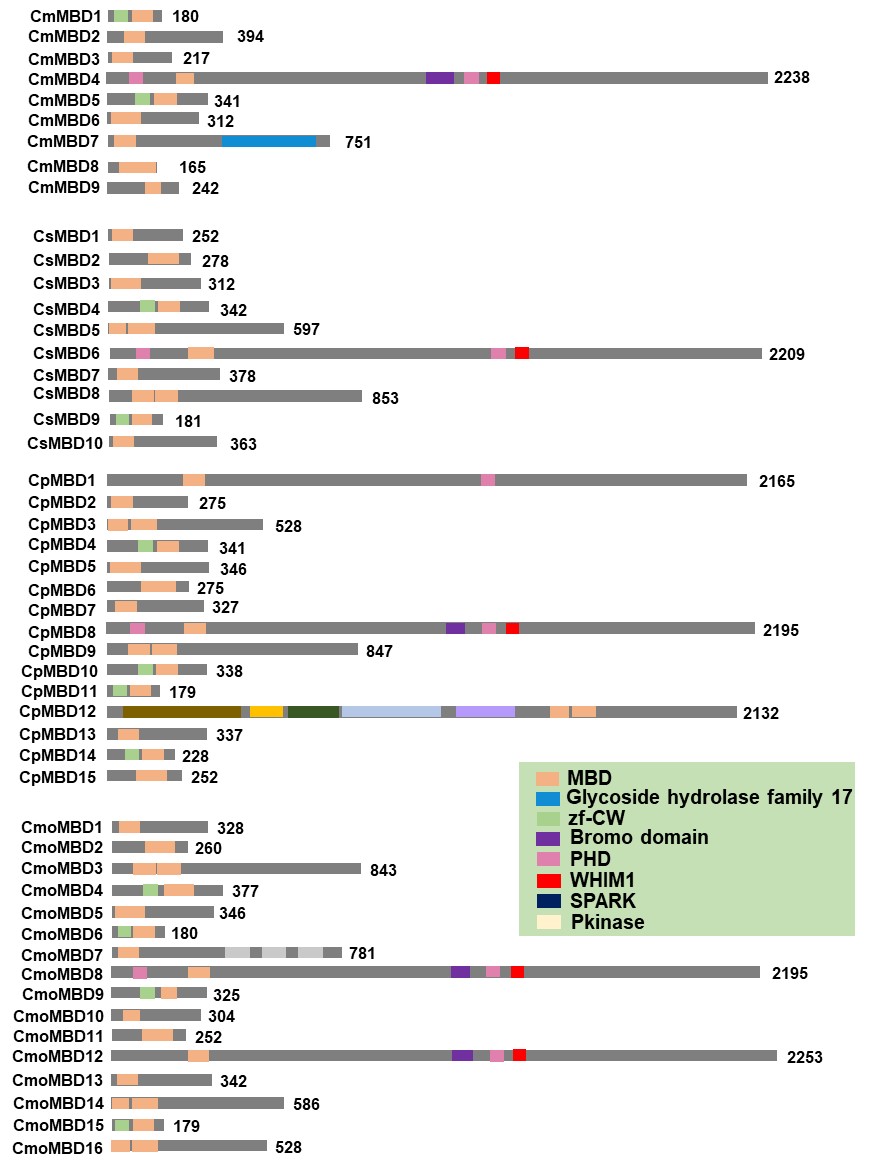

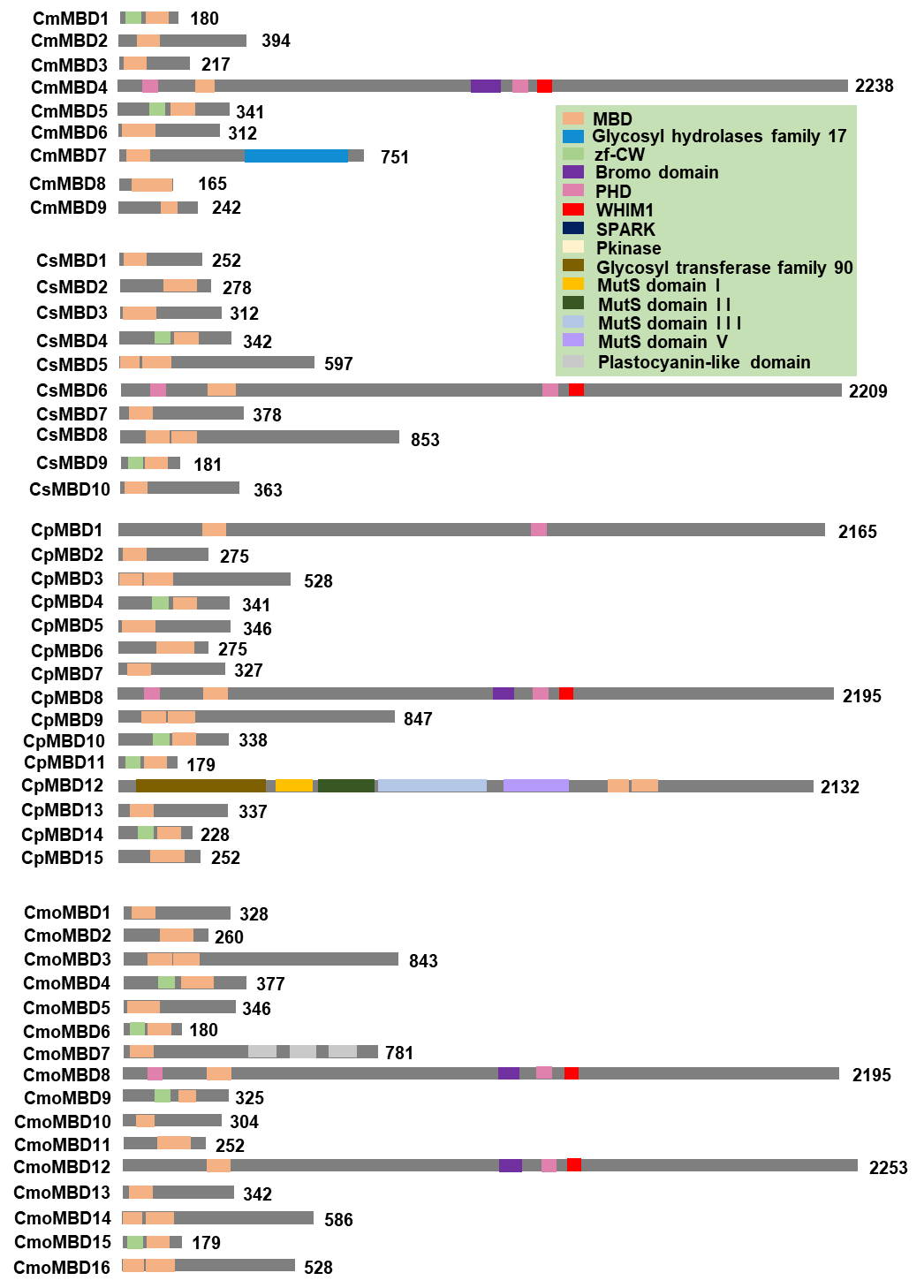
**

**Supplementary Figure S5 |** Conserved domains and their organization in the MBD proteins from melon (Cm), cucumber (Cs), zucchini (Cp), and pumpkin (Cmo).

**
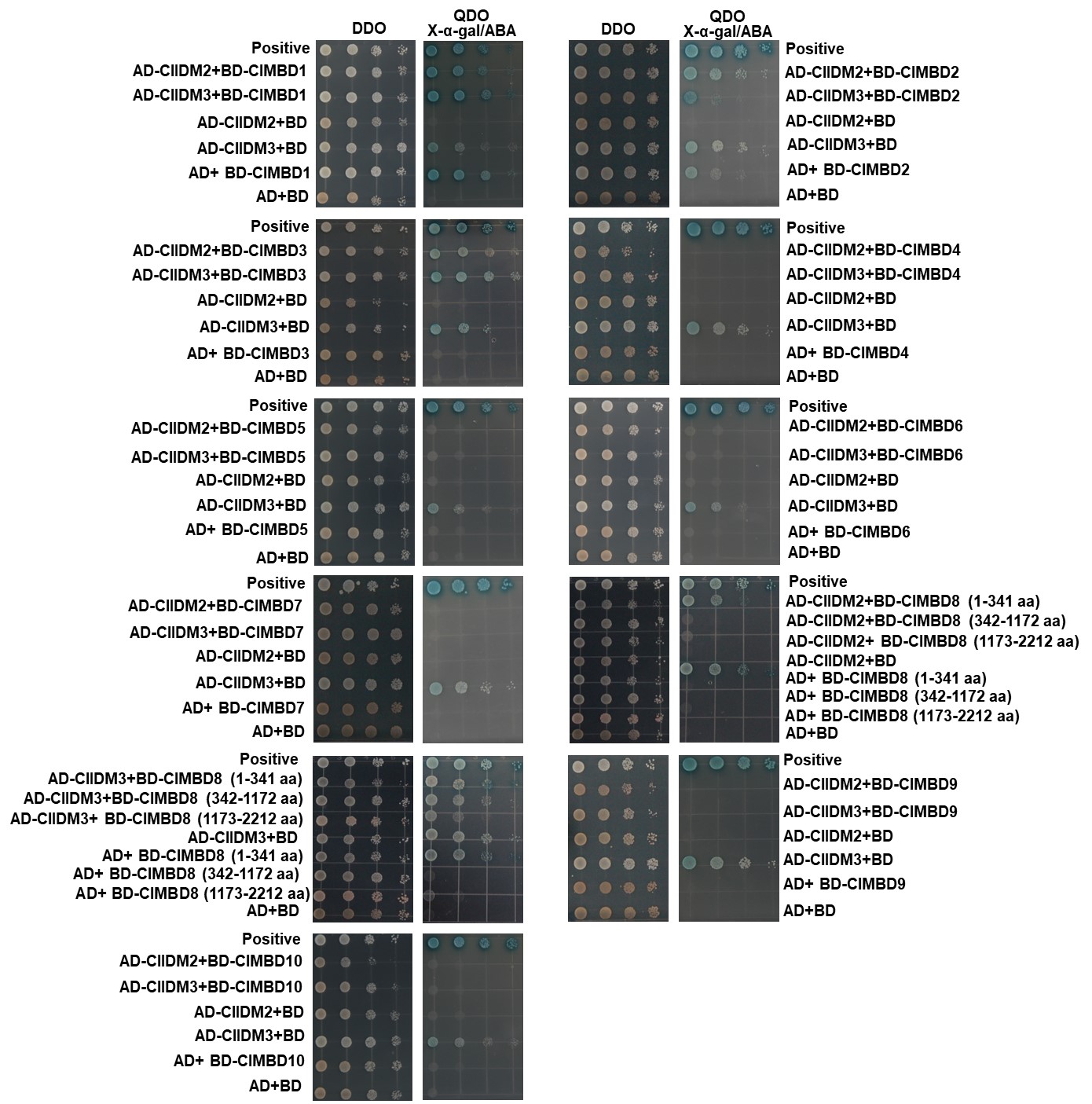
**

**Supplementary Figure S6 |** Putative interactions of ClMBDs with ClIDM2 and ClIDM3. Yeasts harboring the indicated plasmid combinations were grown on selective medium SD/ Trp−His− and β-galactosidase activity showing positive interactions was examined by addition of X-α-gal. Positive control, pGADT7-T + pGBKT7-53; negative control, AD (pGADT7-T) + BD (pGBKT7-Lam). Repeated experiments showed similar results.

**
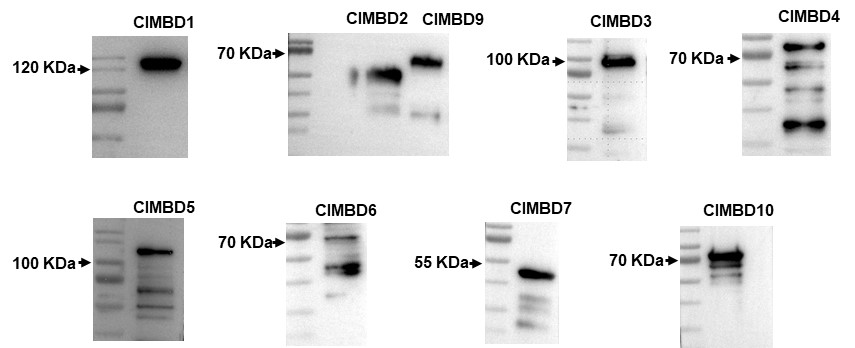
**

**Supplementary Figure S7 |** SDS-PAGE detection of purified ClMBD-GST proteins.

**
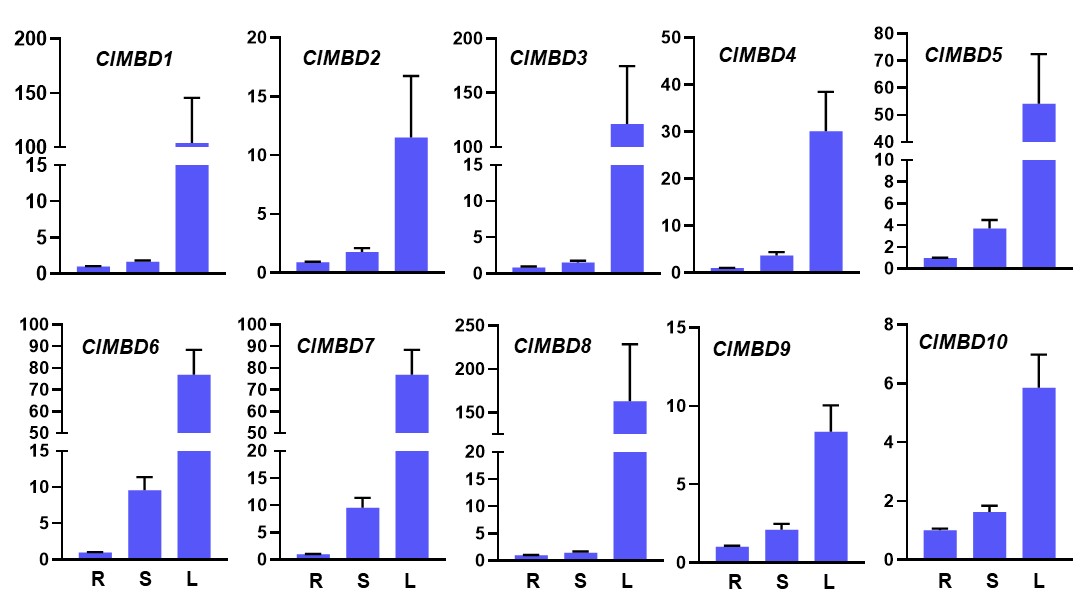
**

**Supplementary Figure S8 |** Expression patterns of the watermelon *ClMBD* genes in root, stem and leaf tissues. Root, stem and leaf samples were collected from four-week-old watermelon plants and qRT-PCR analyses were performed with the watermelon *ClGAPDH* gene as an internal control. Relative expression of the *ClMBD* genes was calculated using the 2^-ΔΔCT^ method. R, root; S, stem, and L, leaf. Experiments were repeated for three times and the data presented are the means ± SE from three independent experiments.

**
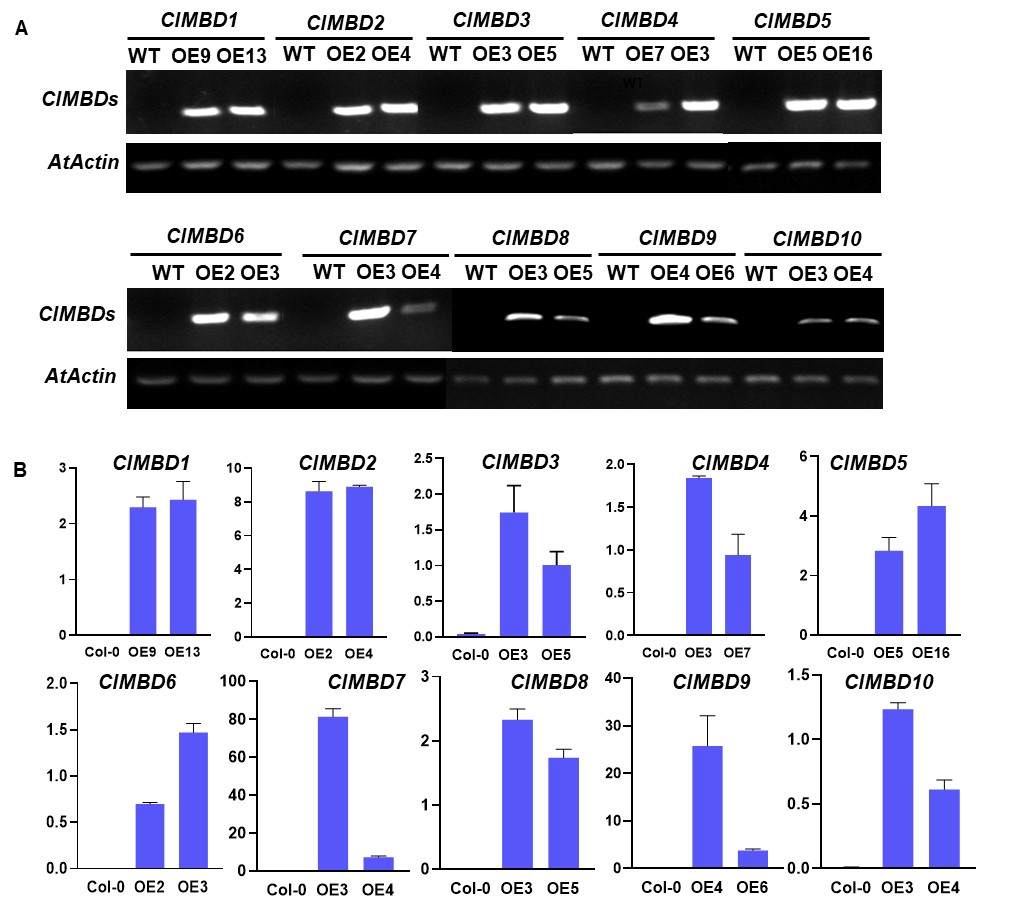
**

**Supplementary Figure S9 |** Transcript levels of the *ClMBD* genes in their corresponding transgenic Arabidopsis lines. Leaf samples were collected from four-week-old Arabidopsis plants, and semi-RT-PCR (**A**) and qRT-PCR (**B**) analyses were performed with the Arabidopsis *AtActin* gene as an internal control. Experiments were repeated for three times and the data presented are the means ± SE from three independent experiments.

**
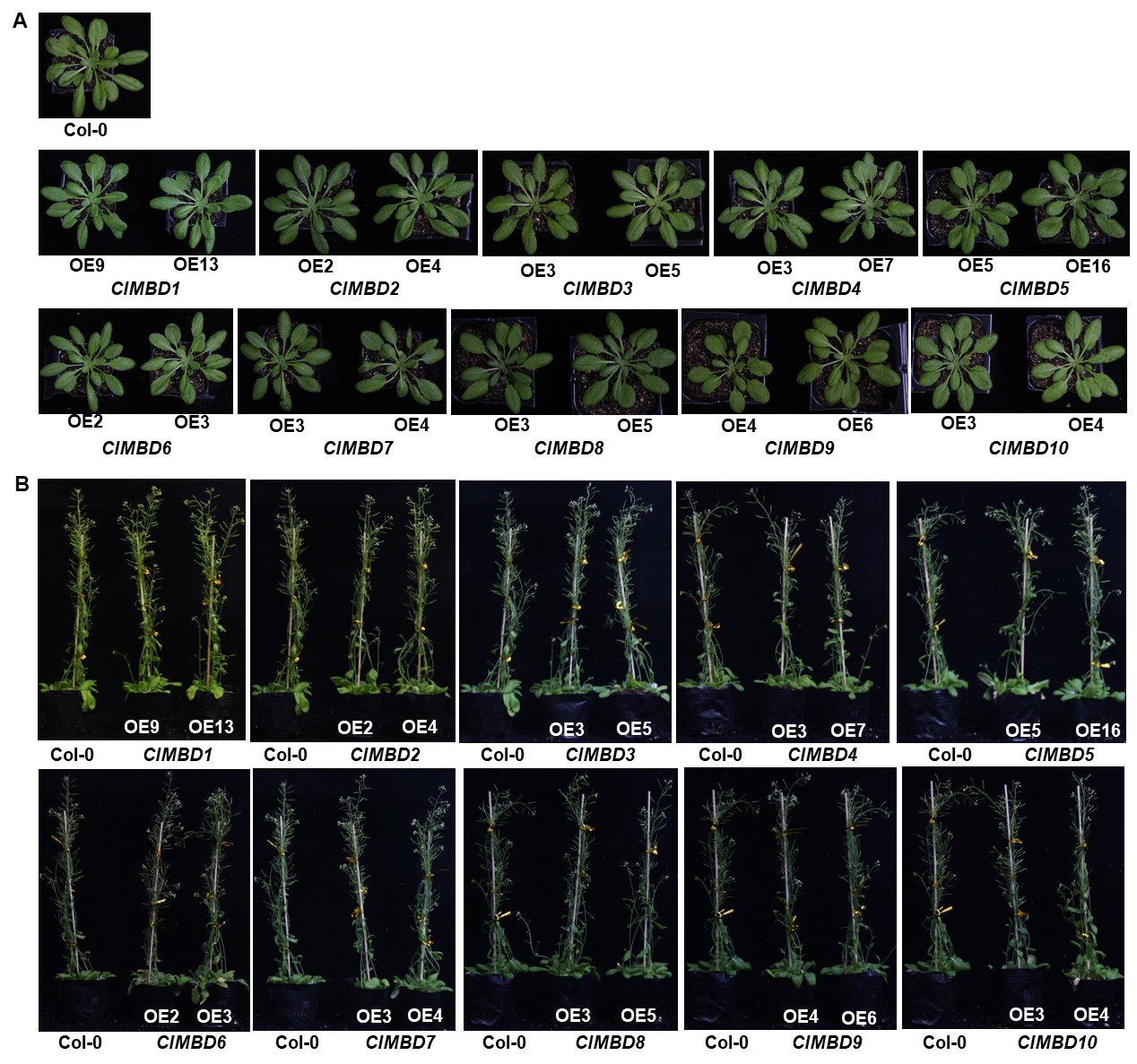
**

**Supplementary Figure S10 |** Morphological comparison the *ClMBDs*-overexpressing Arabidopsis plants with the Col-0 WT plants. (**A**) four-week-old plants; (**B**) six-week-old plants. Repeated experiments showed similar results.

**
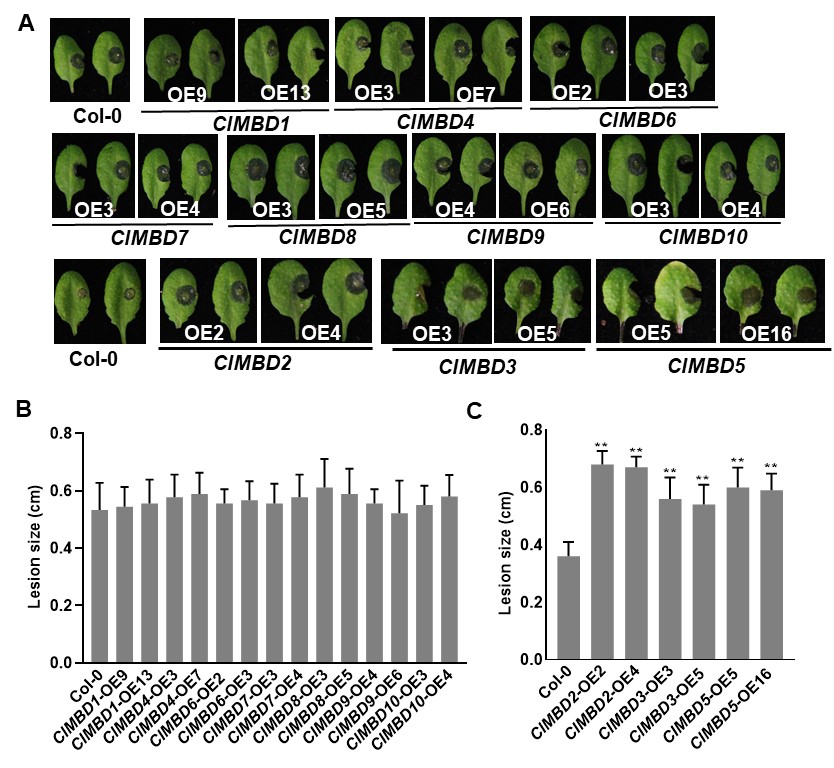
**

**Supplementary Figure S11 |** *Botrytis cinerea*-caused disease phenotype on leaves from the *ClMBDs*-overexpressing and Col-0 WT Arabidopsis plants in detached leaf inoculation assays. (**A**) Disease symptom and (**B,C**) lesion size. *B. cinerea* inoculation was done by dropping spore suspension (1 × 10^5^ spores/mL) on detached leaves and lesion sizes were measured at 3 days after inoculation. Data presented in (**A,B,C**) are repeated for three times with similar results, and results from one representative experiment are shown. Data presented in (**B,C**) are the means ± SD and ** above the columns indicate significant differences at *p* < 0.01 level.

**
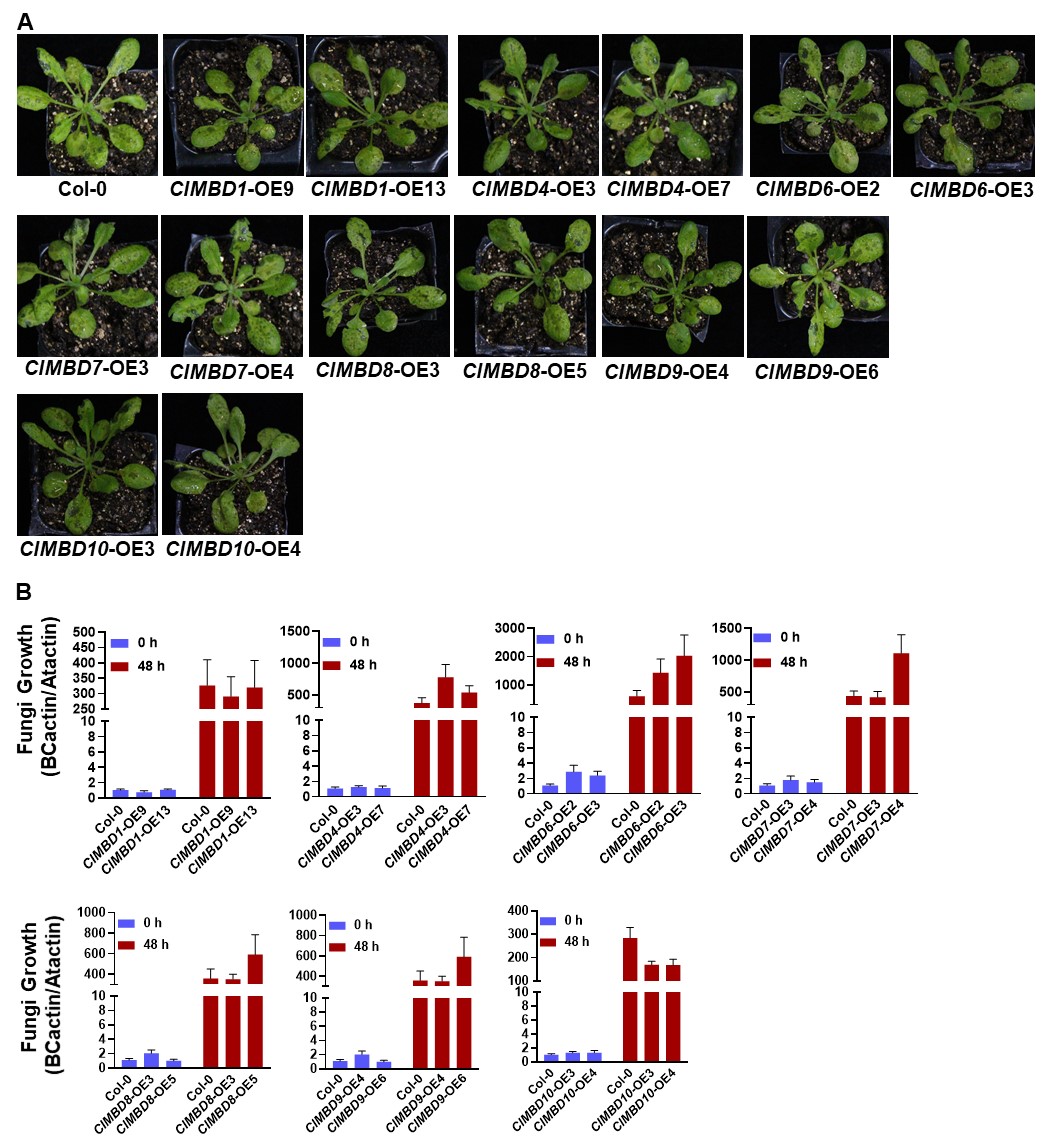
**

**Supplementary Figure S12 |** *Botrytis cinerea*-caused disease phenotype on leaves from the *ClMBDs*-overexpressing and Col-0 WT Arabidopsis plants in whole plant inoculation assays. (**A**) *B. cinerea*-caused disease symptom. Four-week-old plants were inoculated by foliar spraying with *B. cinerea* spore suspension (2 × 10^5^ spores/mL) and photos were taken at 3 d after inoculation. (**B**) *In planta* fungal growth in inoculated plants. Fungal growth was shown as ratios of the transcript level of *B. cinerea BcActinA* to the transcript level of Arabidopsis *AtActin*. Experiments in (**A**) were repeated for three times with similar results, and results from one representative experiment are shown. Data presented in (**B**) are the means ± SE from three independent experiments.

**
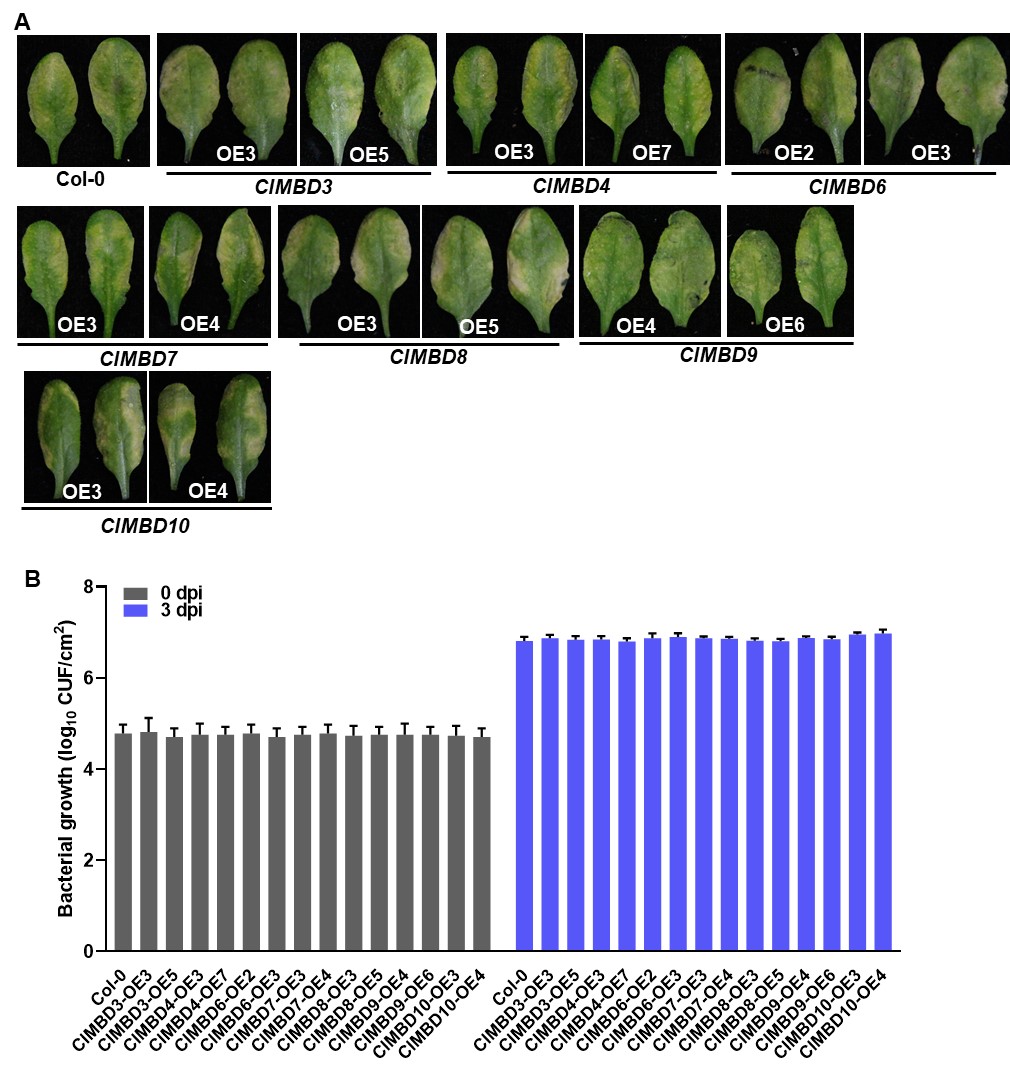
**

**Supplementary Figure S13 |** *Pseudomonas syringae* pv. *tomato* DC3000-caused disease phenotype on leaves from the *ClMBDs*-overexpressing and Col-0 WT Arabidopsis plants. (**A**) *P. syringae* pv. *tomato* DC3000-caused disease symptom. Four-week-old plants were inoculated by injecting with *P. syringae* pv. *tomato* DC3000 bacterial suspension (OD_600_=0.0002) and photos were taken at 72 h after inoculation. (**B**) *In planta* bacterial growth in inoculated leaves. Leaf samples were collected at 0 and 3 d after inoculation and bacterial growth in CFU/cm^2^ leaf area are shown. Experiments in (**A,B**) were repeated for three times with similar results, and results from one representative experiment are shown. Data presented in (**B**) are the means ± SD.

**Supplementary Table S1.** Primers used in this study.

| **Primers** | **Sequences (5’-3’)** |  |
| --- | --- | --- |
| ***Cloning and subcellular localization*** | |  |
| *ClMBD1*-GFP-F | AGCTTTCGCGAGCTCGGTACCATGGTGGCTAAGGGTTCGCC |  |
| *ClMBD1*-GFP-R | GCCCTTGCTCACCATGGTACCCCTTCCCCTAAAAGATCTGTTTCTA |  |
| *ClMBD2*-GFP-F | AGCTTTCGCGAGCTCGGTACCATGTCTGCTTCTGGAACTTCGTT |  |
| *ClMBD2*-GFP-R | GCCCTTGCTCACCATGGTACCAACTTTCGATCGGCCAAGG |  |
| *ClMBD3*-GFP-F | AGCTTTCGCGAGCTCGGTACCATGATGAACCAGAATTCAGAAGATTG |  |
| *ClMBD3*-GFP-R | GCCCTTGCTCACCATGGTACCGTGCCTTTGGAACTCGTTTCTG |  |
| *ClMBD4*-GFP-F | AGCTTTCGCGAGCTCGGTACCATGGCGAGCACACTGGAAGA |  |
| *ClMBD4*-GFP-R | GCCCTTGCTCACCATGGTACCACTTTCTACCTTTTCATCATGTTCCTC |  |
| *ClMBD5*-GFP-F | AGCTTTCGCGAGCTCGGTACCATGGAGAGCAAAGAGGAAGTTATGA |  |
| *ClMBD5*-GFP-R | GCCCTTGCTCACCATGGTACCTGATGCAAGAATCAGCATCAGAA |  |
| *ClMBD6*-GFP-F | AGCTTTCGCGAGCTCGGTACCATGGAAAACGAAGTGGCGC |  |
| *ClMBD6*-GFP-R | GCCCTTGCTCACCATGGTACCCTTACTGATTGCAGTCACCTCGC |  |
| *ClMBD7*-GFP-F | AGCTTTCGCGAGCTCGGTACCATGGCCGCCGCCAGGCCT |  |
| *ClMBD7-*GFP-R | GCCCTTGCTCACCATGGTACCGTATGGGAAGGAAGATTCATTCTTTG |  |
| *ClMBD8*-GFP-F | AGCTTTCGCGAGCTCGGTACCATGATGGAACTCGCCGATTCC |  |
| *ClMBD8*-GFP-R | GCCCTTGCTCACCATGGTACCACCTTCTGGTTCCTTTCTTTTCTTG |  |
| *ClMBD9*-GFP-F | AGCTTTCGCGAGCTCGGTACCATGGCGGCATCAGTGGAGA |  |
| *ClMBD9*-GFP-R | GCCCTTGCTCACCATGGTACCAGGCTTTACCTCATCAGCACCA |  |
| *ClMBD10*-GFP-F | AGCTTTCGCGAGCTCGGTACCATGGATGGTGGTGAAGAAAATGA |  |
| *ClMBD10*-GFP-R | GCCCTTGCTCACCATGGTACCCTTTTCATATTGATCAAAGCAACGT |  |
| ***qRT-PCR*** | |  |
| q*ClMBD1*-F | AAACCAAAGGATGGGGGCGACG |  |
| q*ClMBD1*-R | TCCCTGTTGGCATTTCCTCACC |  |
| q*ClMBD2*-F | CTCTCAACTCTTACAATCTCCC |  |
| q*ClMBD2*-R | CTCTATCTTCAACAACCCAGCCC |  |
| q*ClMBD3*-F | TAATGACATAAACTTGCCTCGCC |  |
| q*ClMBD3*-R | CTTGCTTATCAGACTCCTCACAT |  |
| q*ClMBD4*-F | AGAAAGCAAAGGCAACTCCC |  |
| q*ClMBD4*-R | CTTATGAACTACTGCCTCCTTG |  |
| q*ClMBD5*-F | CATCAGTATGGACGAAACAAC |  |
| q*ClMBD5*-R | GGTGTTACTGTTTCTGCTACTTC |  |
| q*ClMBD6*-F | TTTGATTGGAGTACAGGCGAGAC |  |
| q*ClMBD6*-R | CTCTTCCTGTGGTGGAGTTGCC |  |
| q*ClMBD7*-F | GAAGAACCCTTTACTTGCCATAG |  |
| q*ClMBD7*-R | GTCTTTGGGATGTTGGGTTTGTC |  |
| q*ClMBD8*-F | TCCACGACAACCCTCCGCCCTC |  |
| q*ClMBD8*-R | CGCTCGCATCCGTCACACACC |  |
| q*ClMBD9*-F | ACTGCGAATGAGGAGGTTGTGTC |  |
| q*ClMBD9*-R | CTGTTGGAGTTGTGAAGATGACC |  |
| q*ClMBD10*-F | GCTGTTTCTTCCTCTTCATC |  |
| q*ClMBD10*-R | CTCCATCATTAGCAGAAGGG |  |
| q*ClGAPDH*-F | ATGGGCAAAGTTAAGATCGGCATCA |  |
| q*ClGAPDH*-R | CCAATTCGATATCATCACTCTGC |  |
| q*AtActin*-F | TTGCGACAATGGAACTGGAATG |  |
| q*AtActin*-R | AGCATCCTTTTGCCCCATCC |  |
| q*AtPR1*-F | TCGTCTTTGTAGCTCTTGTAGGTG |  |
| q*AtPR1*-R | TAGATTCTCGTAATCTCAGCTCT |  |
| q*AtPDF1.2*-F | GCTGCTTTTGAAGCACCAACAATG |  |
| q*AtPDF1.2*-R | GCAAGATCCATGTTTTGCTCCCTC |  |
| q*ClPR1*-F | CTTGAGCTTTGCCATGCTGC |  |
| q*ClPR1-*R | GCGTTGGTTGGCATATTGTCG |  |
| q*ClNPR1*-F | TCTTGGCGGCTAGGAGTTTG |  |
| q*ClNPR1-*R | GTCGTGATGGTGGTGGTGAT |  |
| q*ClJAZ1*-F | GCCAACAACTCACCATCTTC |  |
| q*ClJAZ1-*R | CTTGAACCCTATGCTTCCTCT |  |
| q*BcActin*-F | TCCAAGCGTGGTATTCTTACCC |  |
| q*BcActin*-R | TGGTGCTACACGAAGTTCGTTG |  |
| q*AtBEE1*-F | GAAGAGGCCAAGCCACTGAT |  |
| q*AtBEE1*-R | GCATCGTAGCCATTCCCATA |  |
| q*AtbZIP34*-F | GCTTCAACGACGATAACAAA |  |
| q*AtbZIP34*-R | GTACCATCTTCTGGCTCCAT |  |
| q*AtWRKY30*-F | ATGCACGTATAGAAAGTCTCA |  |
| q*AtWRKY30*-R | AAGAATGTATTCCTCGGTAA |  |
| q*AT5G52190*-F | ATCTTCCTCTACGGTGTTGG |  |
| q*AT5G52190*-R | GATGAGATTGGAGGTGTTGTC |  |
| q*AtMAF5*-F | TGGAAGAGCAGCTCAAGAGT |  |
| q*AtMAF5*-R | TCACAAGCTCCATCAATAAC |  |
| q*AtMLO6*-F | TTGTCGAGATCAACCCTGTG |  |
| q*AtMLO6*-R | GGGAATGAACGGTAGCCAGAGG |  |
| q*AtARCK1*-F | TCACCGCGACATTAAACCTG |  |
| q*AtARCK1*-R | TCCTCGCCAATTCAAATCCCAC |  |
| q*AtNAC019*-F | TTACCCGACCGATGAAGAGC |  |
| q*AtNAC019*-R | TTCGGCGATGAGCTGAAGAG |  |
| q*AtERF54*-F | GGGCACGTCTTTGGCTTGG |  |
| q*AtERF54*-R | AAGGCTTGGCGGTCGTAGGC |  |
| q*AtWRKY18*-F | TGCCTACTGAAACATCGGACAC |  |
| q*AtWRKY18*-R | GACGGTGCAAACGAGCATC |  |
| ***Semi-RT-PCR*** | | |
| r*ClMBD1*-F | GAAAGCAGATGCAGATGTTC | |
| r*ClMBD1*-R | AGGAAATTATATTTGATGCCAG | |
| r*ClMBD2*-F | GATCCGACCCGACCCGATTTAC | |
| r*ClMBD2*-R | CGTTTCCTTAATGTTCCTGTTTC | |
| r*ClMBD3*-F | TAAAACAGGTCGCCGAGCAC | |
| r*ClMBD3*-R | CGTTTGGAGTAAAAGCAGTGG | |
| r*ClMBD4*-F | AACGGCCTAAGAAACGAAC | |
| r*ClMBD4*-R | CACCTTGATTTCTCCTGATATG | |
| r*ClMBD5*-F | TGATGTTGAGCTGTGGGAGG | |
| r*ClMBD5*-R | CGTAGATTTCGATTGTATGTCC | |
| r*ClMBD6*-F | AGAGCCTAAAAGCGAAGATG | |
| r*ClMBD6*-R | GTGTTCTTCCAGTACCACGG | |
| r*ClMBD7*-F | CGGCTGCCAGTAGGTCTATTTG | |
| r*ClMBD7*-R | GGGAAGGAAGATTCATTCTTTG | |
| r*ClMBD8*-F | TCATGTGGGTAACGAACAGAG | |
| r*ClMBD8*-R | CAAACCTGGTAAACATCACCAG | |
| r*ClMBD9*-F | AGGTCAGCAAGGATAAGTGAAAAG | |
| r*ClMBD9*-R | GAGTGTCAGGTTTGACAGCTTC | |
| r*ClMBD10*-F | ATGGATGGTGGTGAAGAAAATG | |
| r*ClMBD10*-R | TTGTCAATTGCCCAAAGCCTAC | |
| r*AtActin*-F | TATGTATGTGGCTATTCAGGC | |
| r*AtActin*-R | CAATGTTACCGTACAAGTCC | |
| ***Y2H assays*** | |  |
| *ClMBD1*-BD-F | ATGGCCATGGAGGCCGAATTCATGGTGGCTAAGGGTTCGCC |  |
| *ClMBD1*-BD-R | CCGCTGCAGGTCGACGGATCCTCACCTTCCCCTAAAAGATC |  |
| *ClMBD2*-BD-F | ATGGCCATGGAGGCCGAATTCATGTCTGCTTCTGGAACTTCG |  |
| *ClMBD2*-BD-R | CCGCTGCAGGTCGACGGATCCTCAAACTTTCGATCGGCCAAG |  |
| *ClMBD3*-BD-F | ATGGCCATGGAGGCCGAATTCATGATGAACCAGAATTCAGAAG |  |
| *ClMBD3*-BD-R | CCGCTGCAGGTCGACGGATCCTCAGTGCCTTTGGAACTCG |  |
| *ClMBD4*-BD-F | ATGGCCATGGAGGCCGAATTCATGGCGAGCACACTGGAAG |  |
| *ClMBD4*-BD-R | CCGCTGCAGGTCGACGGATCCTTAACTTTCTACCTTTTCATC |  |
| *ClMBD5*-BD-F | ATGGCCATGGAGGCCGAATTCATGGAGAGCAAAGAGGAAG |  |
| *ClMBD5*-BD-R | CCGCTGCAGGTCGACGGATCCCTATGATGCAAGAATCAGCATC |  |
| *ClMBD6*-BD-F | ATGGCCATGGAGGCCGAATTCATGGAAAACGAAGTGGCGC |  |
| *ClMBD6*-BD-R | CCGCTGCAGGTCGACGGATCCTTACTTACTGATTGCAGTCAC |  |
| *ClMBD7*-BD-F | ATGGCCATGGAGGCCGAATTCATGGCCGCCGCCAGGCCTC |  |
| *ClMBD7-*BD-R | CCGCTGCAGGTCGACGGATCCTCAGTATGGGAAGGAAGATTCA |  |
| *ClMBD8*-BD-F | ATGGCCATGGAGGCCGAATTCATGATGGAACTCGCCGATTCC |  |
| *ClMBD8*-BD-R | CCGCTGCAGGTCGACGGATCCTTAACCTTCTGGTTCCTTTC |  |
| *ClMBD9*-BD-F | ATGGCCATGGAGGCCGAATTCATGGCGGCATCAGTGGAGAAAG |  |
| *ClMBD9*-BD-R | CCGCTGCAGGTCGACGGATCCTCAAGGCTTTACCTCATCAGC |  |
| *ClMBD10*-BD-F | ATGGCCATGGAGGCCGAATTCATGGATGGTGGTGAAGAAAATG |  |
| *ClMBD10*-BD-R | CCGCTGCAGGTCGACGGATCCTTACTTTTCATATTGATCAAAG |  |
| *ClIDM3*-AD-F | GCCATGGAGGCCAGTGAATTCATGGAAGGCCATGGCGATC |  |
| *ClIDM3*-AD-R | CAGCTCGAGCTCGATGGATCCTCACAGTGGTGAGTGGGAATCG |  |
| *ClIDM2*-AD-F | GCCATGGAGGCCAGTGAATTCATGGATGGCAGCTCTCCCC |  |
| *ClIDM2* -AD-R | CAGCTCGAGCTCGATGGATCCTCAGGACTGGAAATCTTTCATCAC |  |
| ***Recombinant protein and purification*** | |  |
| *ClMBD1*-GST-F | GATCTGGTTCCGCGTGGATCCATGGTGGCTAAGGGTTCGCC |  |
| *ClMBD1*-GST-R | GATGCGGCCGCTCGAGTCGACTCACCTTCCCCTAAAAGATC |  |
| *ClMBD2*-GST-F | GATCTGGTTCCGCGTGGATCCATGTCTGCTTCTGGAACTTCG |  |
| *ClMBD2*-GST-R | GATGCGGCCGCTCGAGTCGACTCAAACTTTCGATCGGCCAAG |  |
| *ClMBD3*-GST-F | GATCTGGTTCCGCGTGGATCCATGATGAACCAGAATTCAGAAG |  |
| *ClMBD3*-GST-R | GATGCGGCCGCTCGAGTCGACTCAGTGCCTTTGGAACTCG |  |
| *ClMBD4*-GST-F | GATCTGGTTCCGCGTGGATCCATGGCGAGCACACTGGAAG |  |
| *ClMBD4*-GST-R | GATGCGGCCGCTCGAGTCGACTTAACTTTCTACCTTTTCATC |  |
| *ClMBD5*-GST-F | GATCTGGTTCCGCGTGGATCCATGGAGAGCAAAGAGGAAG |  |
| *ClMBD5*-GST-R | GATGCGGCCGCTCGAGTCGACCTATGATGCAAGAATCAGCATC |  |
| *ClMBD6*-GST-F | GATCTGGTTCCGCGTGGATCCATGGAAAACGAAGTGGCGC |  |
| *ClMBD6*-GST-R | GATGCGGCCGCTCGAGTCGACTTACTTACTGATTGCAGTCAC |  |
| *ClMBD7*-GST-F | GATCTGGTTCCGCGTGGATCCATGGCCGCCGCCAGGCCTC |  |
| *ClMBD7*-GST-R | GATGCGGCCGCTCGAGTCGACTCAGTATGGGAAGGAAGATTCA |  |
| *ClMBD9*-GST-F | GATCTGGTTCCGCGTGGATCCATGGCGGCATCAGTGGAGAAAG |  |
| *ClMBD9*-GST-R | GATGCGGCCGCTCGAGTCGACTCAAGGCTTTACCTCATCAGC |  |
| *ClMBD10*-GST-F | GATCTGGTTCCGCGTGGATCCATGGATGGTGGTGAAGAAAATG |  |
| *ClMBD10*-GST-R | GATGCGGCCGCTCGAGTCGACTTACTTTTCATATTGATCAAAG |  |
| ***BiFC assays*** | |  |
| *ClMBD2*-P2Y-F | CCCTTAATTAACATGTCTGCTTCTGGAACTTCG |  |
| *ClMBD2*-P2Y-R | GGGACTAGTAACTTTCGATCGGCCAAG |  |
| *ClMBD3*-P2Y-F | CCCTTAATTAACATGATGAACCAGAATTCAGAAG |  |
| *ClMBD3*-P2Y-R | GGGACTAGTGTGCCTTTGGAACTCG |  |
| *ClIDM2*-P2Y-1F | CCCTTAATTAACATGGATGGCAGCTCTCCCC |  |
| *ClIDM2*-P2Y-1R | GGGACTAGTGGACTGGAAATCTTTCATCAC |  |
| *ClIDM3*-P2Y-1F | CCCTTAATTAACATGGAAGGCCATGGCGATC |  |
| *ClIDM3*-P2Y-1R | GGGACTAGTCAGTGGTGAGTGGGAATCG |  |

**Supplementary Table S2.** *MBD* genes in *cucurbit* crops.

| Species | Genes | Loci | ORF (bp) | Size (aa) | MW (KDa) | *p*I |
| --- | --- | --- | --- | --- | --- | --- |
| *Cucumis melo* | CmMBD1 | MELO3C015875.2.1 | 543 | 180 | 20.60 | 8.83 |
|  | CmMBD2 | MELO3C023419.2.1 | 1185 | 394 | 44.81 | 5.46 |
|  | CmMBD3 | MELO3C010329.2.1 | 654 | 217 | 24.43 | 5.51 |
|  | CmMBD4 | MELO3C009946.2.1 | 6717 | 2238 | 247.49 | 5.25 |
|  | CmMBD5 | MELO3C003942.2.1 | 1026 | 341 | 38.21 | 5.82 |
|  | CmMBD6 | MELO3C004492.2.1 | 939 | 312 | 34.80 | 4.88 |
|  | CmMBD7 | MELO3C017880.2.1 | 2256 | 751 | 82.43 | 4.89 |
|  | CmMBD8 | MELO3C033074.2.1 | 498 | 165 | 18.90 | 9.69 |
|  | CmMBD9 | MELO3C020629.2.1 | 729 | 242 | 26.03 | 7.20 |
| *Cucumis sativus* | CsMBD1 | CsaV3_1G030350.1 | 759 | 252 | 28.61 | 5.37 |
|  | CsMBD2 | CsaV3_1G034280.1 | 837 | 278 | 30.56 | 9.32 |
|  | CsMBD3 | CsaV3_2G002970.1 | 939 | 312 | 34.89 | 4.83 |
|  | CsMBD4 | CsaV3_2G009010.1 | 1029 | 342 | 38.29 | 5.81 |
|  | CsMBD5 | CsaV3_3G017000.1 | 1794 | 597 | 66.43 | 8.52 |
|  | CsMBD6 | CsaV3_3G042270.1 | 6630 | 2209 | 244.68 | 5.21 |
|  | CsMBD7 | CsaV3_4G001590.1 | 1137 | 378 | 41.31 | 4.60 |
|  | CsMBD8 | CsaV3_5G003080.1 | 2562 | 853 | 95.26 | 8.10 |
|  | CsMBD9 | CsaV3_7G028470.1 | 546 | 181 | 20.50 | 8.28 |
|  | CsMBD10 | CsaV3_7G032420.1 | 1092 | 363 | 40.64 | 4.71 |
| *Cucurbita pepo* | CpMBD1 | Cp4.1LG03g07700.1 | 6498 | 2165 | 240.25 | 5.52 |
|  | CpMBD2 | Cp4.1LG03g13750.1 | 828 | 275 | 30.38 | 4.56 |
|  | CpMBD3 | Cp4.1LG09g00820.1 | 1587 | 528 | 59.11 | 7.57 |
|  | CpMBD4 | Cp4.1LG11g06750.1 | 1026 | 341 | 38.47 | 5.85 |
|  | CpMBD5 | Cp4.1LG11g10530.1 | 1041 | 346 | 38.89 | 4.62 |
|  | CpMBD6 | Cp4.1LG02g13350.1 | 828 | 275 | 29.93 | 9.04 |
|  | CpMBD7 | Cp4.1LG02g14780.1 | 984 | 327 | 35.85 | 4.59 |
|  | CpMBD8 | Cp4.1LG04g05220.1 | 6588 | 2195 | 243.26 | 5.21 |
|  | CpMBD9 | Cp4.1LG05g03710.1 | 2544 | 847 | 94.49 | 8.64 |
|  | CpMBD10 | Cp4.1LG07g07630.1 | 1017 | 338 | 37.64 | 8.38 |
|  | CpMBD11 | Cp4.1LG12g11910.1 | 540 | 179 | 20.58 | 8.30 |
|  | CpMBD12 | Cp4.1LG14g06090.1 | 6396 | 2132 | 240.69 | 7.21 |
|  | CpMBD13 | Cp4.1LG17g08950.1 | 1014 | 337 | 37.39 | 4.91 |
|  | CpMBD14 | Cp4.1LG17g06780.1 | 687 | 228 | 26.00 | 9.04 |
|  | CpMBD15 | Cp4.1LG20g08550.1 | 759 | 252 | 27.86 | 9.47 |
| *Cucurbita moschata* | CmoMBD1 | CmoCh01G001510.1 | 987 | 328 | 35.96 | 4.62 |
|  | CmoMBD2 | CmoCh01G006300.1 | 783 | 260 | 28.41 | 9.21 |
|  | CmoMBD3 | CmoCh02G015110.1 | 2532 | 843 | 94.03 | 8.61 |
|  | CmoMBD4 | CmoCh05G007650.1 | 1134 | 377 | 42.57 | 5.57 |
|  | CmoMBD5 | CmoCh05G013070.1 | 1041 | 346 | 38.89 | 4.66 |
|  | CmoMBD6 | CmoCh08G003660.1 | 543 | 180 | 20.52 | 8.83 |
|  | CmoMBD7 | CmoCh08G006050.1 | 2346 | 781 | 84.07 | 4.80 |
|  | CmoMBD8 | CmoCh11G016990.1 | 6588 | 2195 | 243.29 | 5.21 |
|  | CmoMBD9 | CmoCh12G009280.1 | 978 | 325 | 36.26 | 6.47 |
|  | CmoMBD10 | CmoCh12G012290.1 | 915 | 304 | 34.58 | 5.18 |
|  | CmoMBD11 | CmoCh13G001230.1 | 759 | 252 | 27.89 | 9.56 |
|  | CmoMBD12 | CmoCh14G006530.1 | 6762 | 2253 | 249.91 | 5.53 |
|  | CmoMBD13 | CmoCh14G013740.1 | 1029 | 342 | 37.30 | 4.34 |
|  | CmoMBD14 | CmoCh16G001360.1 | 1761 | 586 | 65.66 | 6.91 |
|  | CmoMBD15 | CmoCh17G011260.1 | 540 | 179 | 20.55 | 8.30 |
|  | CmoMBD16 | CmoCh18G012640.1 | 1587 | 528 | 59.07 | 6.79 |

**Supplementary Table S3.** Ka/Ks ratios of the *ClMBD* gene pairs.

| Gene pairs | Ka | Ks | Ka/Ks | Types of selection |
| --- | --- | --- | --- | --- |
| *ClMBD1*-*ClMBD3* | 0.6802 | 1.8084 | 0.3761 | Purifying selection |
| *ClMBD4*-*ClMBD5* | 0.6829 | NA | NA | NA |
| *ClMBD4*-*ClMBD6* | 0.6495 | 2.5244 | 0.2573 | Purifying selection |
| *ClMBD4*-*ClMBD9* | 0.6956 | 2.2990 | 0.3026 | Purifying selection |
| *ClMBD5*-*ClMBD6* | 0.5990 | 1.5916 | 0.3763 | Purifying selection |
| *ClMBD5*-*ClMBD9* | 0.8227 | 2.0203 | 0.4072 | Purifying selection |
| *ClMBD6*-*ClMBD9* | 0.6767 | 1.6544 | 0.4090 | Purifying selection |

**Supplementary Table S4.** The number of colinear gene pairs between watermelon (*Citrullus lanatus*) and *Arabidopsis thaliana*, melon (*Cucumis melon*), cucumber (*Cucumis sativus*), pumpkin (*Cucurbita moshata*), zucchini (*Cucurbita pepo*).

| **chromosome** | **genes** | **chromosome** | **genes** |
| --- | --- | --- | --- |
| ***Citrullus lanatus*** | | ***Arabidopsis thaliana*** | |
| Chr01 | Cla97C01G003060.1 | Chr5 | AT5G52230.1 |
| Chr03 | Cla97C03G052410.1 | Chr3 | AT3G46580.1 |
| Chr03 | Cla97C03G052410.1 | Chr5 | AT5G59380.1 |
| Chr06 | Cla97C06G120480.1 | Chr1 | AT1G15340.1 |
| Chr06 | Cla97C06G120480.1 | Chr3 | AT3G15790.1 |
| Chr09 | Cla97C09G165060.1 | Chr1 | AT1G15340.1 |
| Chr09 | Cla97C09G165060.1 | Chr3 | AT3G15790.1 |
| Chr10 | Cla97C10G197170.1 | Chr3 | AT3G01460.1 |
| Chr11 | Cla97C11G209600.1 | Chr3 | AT3G15790.1 |
| ***Citrullus lanatus*** | | ***Cucumis melon*** | |
| Chr03 | Cla97C03G052410.1 | Chr12 | MELO3C020629.2.1 |
| Chr06 | Cla97C06G120480.1 | Chr01 | MELO3C023419.2.1 |
| Chr06 | Cla97C06G120480.1 | Chr02 | MELO3C010329.2.1 |
| Chr06 | Cla97C06G120480.1 | Chr05 | MELO3C004492.2.1 |
| Chr06 | Cla97C06G120480.1 | Chr07 | MELO3C017880.2.1 |
| Chr07 | Cla97C07G139410.1 | Chr07 | MELO3C017880.2.1 |
| Chr09 | Cla97C09G165060.1 | Chr01 | MELO3C023419.2.1 |
| Chr09 | Cla97C09G165060.1 | Chr05 | MELO3C004492.2.1 |
| Chr09 | Cla97C09G165060.1 | Chr07 | MELO3C017880.2.1 |
| Chr09 | Cla97C09G169310.1 | Chr01 | MELO3C015875.2.1 |
| Chr10 | Cla97C10G197170.1 | Chr04 | MELO3C009946.2.1 |
| Chr11 | Cla97C11G209600.1 | Chr01 | MELO3C023419.2.1 |
| Chr11 | Cla97C11G209600.1 | Chr02 | MELO3C010329.2.1 |
| Chr11 | Cla97C11G209600.1 | Chr05 | MELO3C004492.2.1 |
| Chr11 | Cla97C11G217560.1 | Chr05 | MELO3C003942.2.1 |
| ***Citrullus lanatus*** | | ***Cucumis sativus*** | |
| Chr01 | Cla97C01G003060.1 | Chr03 | CsaV3_3G017000.1 |
| Chr01 | Cla97C01G003060.1 | Chr05 | CsaV3_5G003080.1 |
| Chr03 | Cla97C03G052410.1 | Chr01 | CsaV3_1G034280.1 |
| Chr05 | Cla97C05G089970.1 | Chr03 | CsaV3_3G017000.1 |
| Chr05 | Cla97C05G089970.1 | Chr05 | CsaV3_5G003080.1 |
| Chr06 | Cla97C06G120480.1 | Chr01 | CsaV3_1G030350.1 |
| Chr06 | Cla97C06G120480.1 | Chr02 | CsaV3_2G002970.1 |
| Chr06 | Cla97C06G120480.1 | Chr04 | CsaV3_4G001590.1 |
| Chr06 | Cla97C06G120480.1 | Chr07 | CsaV3_7G032420.1 |
| Chr07 | Cla97C07G139410.1 | Chr04 | CsaV3_4G001590.1 |
| Chr09 | Cla97C09G165060.1 | Chr02 | CsaV3_2G002970.1 |
| Chr09 | Cla97C09G165060.1 | Chr04 | CsaV3_4G001590.1 |
| Chr09 | Cla97C09G165060.1 | Chr07 | CsaV3_7G032420.1 |
| Chr09 | Cla97C09G169310.1 | Chr07 | CsaV3_7G028470.1 |
| Chr10 | Cla97C10G197170.1 | Chr03 | CsaV3_3G042270.1 |
| Chr11 | Cla97C11G209600.1 | Chr01 | CsaV3_1G030350.1 |
| Chr11 | Cla97C11G209600.1 | Chr02 | CsaV3_2G002970.1 |
| Chr11 | Cla97C11G209600.1 | Chr04 | CsaV3_4G001590.1 |
| Chr11 | Cla97C11G209600.1 | Chr07 | CsaV3_7G032420.1 |
| Chr11 | Cla97C11G217560.1 | Chr02 | CsaV3_2G009010.1 |
| ***Citrullus lanatus*** | | ***Cucurbita moshata*** | |
| Chr01 | Cla97C01G003060.1 | Chr02 | CmoCh02G015110.1 |
| Chr01 | Cla97C01G003060.1 | Chr16 | CmoCh16G001360.1 |
| Chr03 | Cla97C03G052410.1 | Chr01 | CmoCh01G006300.1 |
| Chr03 | Cla97C03G052410.1 | Chr13 | CmoCh13G001230.1 |
| Chr05 | Cla97C05G089970.1 | Chr02 | CmoCh02G015110.1 |
| Chr05 | Cla97C05G089970.1 | Chr16 | CmoCh16G001360.1 |
| Chr05 | Cla97C05G089970.1 | Chr18 | CmoCh18G012640.1 |
| Chr06 | Cla97C06G120480.1 | Chr01 | CmoCh01G001510.1 |
| Chr06 | Cla97C06G120480.1 | Chr05 | CmoCh05G013070.1 |
| Chr06 | Cla97C06G120480.1 | Chr12 | CmoCh12G012290.1 |
| Chr06 | Cla97C06G120480.1 | Chr14 | CmoCh14G013740.1 |
| Chr07 | Cla97C07G139410.1 | Chr01 | CmoCh01G001510.1 |
| Chr07 | Cla97C07G139410.1 | Chr14 | CmoCh14G013740.1 |
| Chr09 | Cla97C09G165060.1 | Chr01 | CmoCh01G001510.1 |
| Chr09 | Cla97C09G165060.1 | Chr05 | CmoCh05G013070.1 |
| Chr09 | Cla97C09G169310.1 | Chr08 | CmoCh08G003660.1 |
| Chr09 | Cla97C09G165060.1 | Chr08 | CmoCh08G006050.1 |
| Chr09 | Cla97C09G165060.1 | Chr12 | CmoCh12G012290.1 |
| Chr09 | Cla97C09G165060.1 | Chr14 | CmoCh14G013740.1 |
| Chr09 | Cla97C09G169310.1 | Chr17 | CmoCh17G011260.1 |
| Chr10 | Cla97C10G197170.1 | Chr11 | CmoCh11G016990.1 |
| Chr10 | Cla97C10G197170.1 | Chr14 | CmoCh14G006530.1 |
| Chr11 | Cla97C11G209600.1 | Chr05 | CmoCh05G013070.1 |
| Chr11 | Cla97C11G217560.1 | Chr05 | CmoCh05G007650.1 |
| Chr11 | Cla97C11G209600.1 | Chr12 | CmoCh12G012290.1 |
| Chr11 | Cla97C11G217560.1 | Chr12 | CmoCh12G009280.1 |
| ***Citrullus lanatus*** | | ***Cucurbita pepo*** | |
| Chr01 | Cla97C01G003060.1 | Chr05 | Cp4.1LG05g03710.1 |
| Chr01 | Cla97C01G003060.1 | Chr14 | Cp4.1LG14g06090.1 |
| Chr03 | Cla97C03G052410.1 | Chr02 | Cp4.1LG02g13350.1 |
| Chr03 | Cla97C03G052410.1 | Chr20 | Cp4.1LG20g08550.1 |
| Chr05 | Cla97C05G089970.1 | Chr05 | Cp4.1LG05g03710.1 |
| Chr05 | Cla97C05G089970.1 | Chr09 | Cp4.1LG09g00820.1 |
| Chr05 | Cla97C05G089970.1 | Chr14 | Cp4.1LG14g06090.1 |
| Chr06 | Cla97C06G120480.1 | Chr02 | Cp4.1LG02g14780.1 |
| Chr06 | Cla97C06G120480.1 | Chr11 | Cp4.1LG11g10530.1 |
| Chr07 | Cla97C07G139410.1 | Chr02 | Cp4.1LG02g14780.1 |
| Chr09 | Cla97C09G165060.1 | Chr02 | Cp4.1LG02g14780.1 |
| Chr09 | Cla97C09G165060.1 | Chr11 | Cp4.1LG11g10530.1 |
| Chr09 | Cla97C09G169310.1 | Chr12 | Cp4.1LG12g11910.1 |
| Chr09 | Cla97C09G165060.1 | Chr17 | Cp4.1LG17g08950.1 |
| Chr10 | Cla97C10G197170.1 | Chr03 | Cp4.1LG03g07700.1 |
| Chr10 | Cla97C10G197170.1 | Chr04 | Cp4.1LG04g05220.1 |
| Chr11 | Cla97C11G217560.1 | Chr07 | Cp4.1LG07g07630.1 |
| Chr11 | Cla97C11G209600.1 | Chr11 | Cp4.1LG11g10530.1 |
| Chr11 | Cla97C11G217560.1 | Chr11 | Cp4.1LG11g06750.1 |

**Supplementary Table S5.** Up-regulated genes in *ClMBD2*-OE2 plants.

| Gene ID | Description | log_2_ FC | *P*-value |
| --- | --- | --- | --- |
| AT5G24780 | AtVSP1 | 3.91 | 0.0020 |
| AT5G24770 | AtVSP2 | 3.65 | 0.0007 |
| AT2G39030 | AtNATA1 | 2.34 | 0.0337 |
| AT3G12910 | NAC transcriptional factor | 2.27 | 0.0132 |
| AT5G24110 | AtWRKY30 | 2.27 | 0.0093 |
| AT1G76930 | AtEXT4 | 2.09 | 0.0293 |
| AT1G07160 | Protein phosphatase 2C | 2.05 | 0.0336 |
| AT2G07671 | ATP synthase subunit C | 2.04 | 0.0005 |
| AT1G43910 | Nucleoside triphosphate hydrolases | 1.91 | 0.0026 |
| AT2G24850 | AtTAT3 | 1.88 | 0.0023 |
| AT4G11470 | AtCRK31 | 1.83 | 0.0149 |
| AT1G52890 | AtNAC019 | 1.75 | 0.0452 |
| AT5G52170 | AtHDG7 | 1.67 | 0.0299 |
| AT4G28140 | AtERF54 | 1.57 | 0.0451 |
| AT1G26380 | fad-linked oxidoreductase 1 | 1.50 | 0.0430 |
| AT3G25780 | AtAOC3 | 1.49 | 0.0173 |
| AT4G21390 | AtB120 | 1.48 | 0.0063 |
| AT1G52400 | AtBGLU18 | 1.45 | 0.0026 |
| AT2G18660 | AtPNP-A | 1.44 | 0.0188 |
| AT2G29110 | AtGLR2.8 | 1.42 | 0.0325 |
| AT4G39830 | Cupredoxin | 1.39 | 0.0255 |
| AT1G14250 | GDA1/CD39 nucleoside phosphatase | 1.36 | 0.0176 |
| AT1G07430 | AtHAI2 | 1.34 | 0.0232 |
| AT4G33467 | hypothetical protein | 1.32 | 0.0149 |
| AT3G26910 | hydroxyproline-rich glycoprotein | 1.30 | 0.0069 |
| AT1G61360 | S-locus lectin protein kinase | 1.27 | 0.0126 |
| AT3G59350 | AtMAZZA | 1.22 | 0.0232 |
| AT5G25930 | Leucine-rich repeat kinase | 1.21 | 0.0195 |
| AT1G61560 | AtMLO6 | 1.21 | 0.0454 |
| AT2G06050 | AtOPR3 | 1.20 | 0.0181 |
| AT3G57260 | AtBGL2 | 1.20 | 0.0305 |
| AT1G21120 | AtIGMT2 | 1.11 | 0.0416 |
| AT3G56570 | SET domain-containing protein | 1.08 | 0.0497 |
| AT3G51450 | Calcium-dependent phosphotriesterase | 1.06 | 0.0128 |
| AT1G71697 | AtCK1 | 1.06 | 0.0293 |
| AT1G51402 | hypothetical protein | 1.04 | 0.0155 |
| AT5G01600 | AtFER1 | 1.03 | 0.0215 |
| AT4G11890 | AtARCK1 | 1.03 | 0.0446 |
| AT2G39240 | Transcription initiation factor RRN3 | 1.01 | 0.0455 |
| AT1G56510 | AtWRR4 | 1.01 | 0.0120 |
| AT4G31800 | AtWRKY18 | 0.96 | 0.0422 |
| AT3G26980 | AtMUB4 | 0.87 | 0.0480 |
| AT1G25220 | AtASB1 | 0.82 | 0.0135 |
| AT3G09830 | PBS1-LIKE 39 | 0.82 | 0.0461 |
| AT4G00970 | AtCRK41 | 0.75 | 0.0462 |
| AT2G13790 | AtSERK4 | 0.68 | 0.0335 |
| AT4G01026 | PYR1-LIKE 7 | 0.64 | 0.0389 |
| AT4G37640 | Calcium atpase 2 | 0.64 | 0.0487 |
| newGene_305 | -- | 3.00 | 0.0002 |
| newGene_603 | -- | 2.14 | 7.63E^-6^ |
| newGene_621 | -- | 1.84 | 9.46E^-6^ |
| newGene_77 | -- | 0.83 | 0.0499 |

**Supplementary Table S6.** Down-regulated genes in *ClMBD2*-OE2 plants.

| Gene ID | gene name | log_2_ FC | *P-*value |
| --- | --- | --- | --- |
| AT5G52190 | Sugar isomerase | -4.44 | 0.0154 |
| AT3G50800 | PADRE protein | -1.96 | 0.0188 |
| AT1G02450 | AtNIMIN1 | -1.83 | 0.0042 |
| AT4G38775 | hypothetical protein | -1.56 | 0.0100 |
| AT5G65080 | AtMAF5 | -1.52 | 0.0095 |
| AT3G54430 | AtSRS6 | -1.48 | 0.0143 |
| AT1G72910 | AtTIR-NBS8 | -1.30 | 0.0070 |
| AT3G61190 | AtBAP1 | -1.27 | 0.0327 |
| AT1G18400 | AtBEE1 | -1.23 | 0.0185 |
| AT3G28345 | AtABCB15 | -1.18 | 0.0480 |
| AT1G03920 | AtNDR2 | -1.13 | 0.0401 |
| AT2G42380 | AtbZIP34 | -1.11 | 0.0377 |
| AT5G17780 | alpha/beta-Hydrolases | -1.10 | 0.0364 |
| AT5G19600 | AtSULTR3;5 | -1.05 | 0.0254 |
| AT2G41370 | AtBOP2 | -0.97 | 0.0390 |
| AT1G22330 | RNA-binding protein | -0.97 | 0.0426 |
| AT5G42720 | Glycosyl hydrolase family 17 protein | -0.90 | 0.0388 |
| AT2G40750 | AtWRKY54 | -0.87 | 0.0312 |

**Supplementary Table S7.** GO terms for up-regulated genes in *ClMBD2*-OE2 plants.

| GO term | Description | Number in input | Number in background |
| --- | --- | --- | --- |
| ***Biological process*** |  |  |  |
| GO:0009987 | cellular process | 19 | 7967 |
| GO:0008152 | metabolic process | 17 | 7048 |
| GO:0044699 | single-organism process | 12 | 5428 |
| GO:0065007 | biological regulation | 7 | 3057 |
| GO:0050896 | response to stimulus | 8 | 2568 |
| GO:0071840 | cellular component organization | 1 | 1716 |
| GO:0051179 | localization | 2 | 1323 |
| GO:0032502 | developmental process | 2 | 1041 |
| GO:0032501 | multicellular organismal process | 4 | 1027 |
| GO:0023052 | signaling | 3 | 893 |
| GO:0022414 | reproductive process | 2 | 669 |
| GO:0051704 | multi-organism process | 5 | 647 |
| GO:0002376 | immune system process | 1 | 175 |
| ***Cellular component*** |  |  |  |
| GO:0005623 | cell | 15 | 7913 |
| GO:0044464 | cell part | 15 | 7913 |
| GO:0016020 | membrane | 16 | 6894 |
| GO:0043226 | organelle | 10 | 6282 |
| GO:0044425 | membrane part | 14 | 5970 |
| GO:0044422 | organelle part | 1 | 2232 |
| GO:0032991 | macromolecular complex | 3 | 2068 |
| GO:0005576 | extracellular region | 2 | 829 |
| ***Molecular function*** |  |  |  |
| GO:0005488 | binding | 33 | 10263 |
| GO:0003824 | catalytic activity | 29 | 9014 |
| GO:0005215 | transporter activity | 3 | 931 |
| GO:0001071 | transcription factor activity | 4 | 821 |
| GO:0005198 | structural molecule activity | 1 | 448 |
| GO:0000988 | transcription factor activity | 1 | 136 |
| GO:0009055 | electron carrier activity | 1 | 133 |
| GO:0060089 | molecular transducer activity | 2 | 104 |
| GO:0004871 | signal transducer activity | 2 | 104 |
| GO:0098772 | molecular function regulator | 1 | 81 |

**Supplementary Table S8.** GO terms for down-regulated genes in *ClMBD2*-OE2 plants.

| GO term | Description | Number in input | Number in background |
| --- | --- | --- | --- |
| ***Biological process*** |  |  |  |
| GO:0009987 | cellular process | 5 | 7967 |
| GO:0008152 | metabolic process | 5 | 7048 |
| GO:0044699 | single-organism process | 4 | 5428 |
| GO:0065007 | biological regulation | 6 | 3057 |
| GO:0050896 | response to stimulus | 5 | 2568 |
| GO:0032502 | developmental process | 1 | 1041 |
| GO:0032501 | multicellular organismal process | 1 | 1027 |
| GO:0023052 | signaling | 3 | 893 |
| GO:0022414 | reproductive process | 1 | 669 |
| GO:0051704 | multi-organism process | 2 | 647 |
| GO:0000003 | reproduction | 1 | 446 |
| GO:0002376 | immune system process | 2 | 175 |
| ***Cellular component*** |  |  |  |
| GO:0005623 | cell | 8 | 7913 |
| GO:0044464 | cell part | 8 | 7913 |
| GO:0016020 | membrane | 6 | 6894 |
| GO:0043226 | organelle | 5 | 6282 |
| GO:0044425 | membrane part | 4 | 5970 |
| GO:0032991 | macromolecular complex | 1 | 2068 |
| ***Molecular function*** |  |  |  |
| GO:0005488 | binding | 10 | 10263 |
| GO:0003824 | catalytic activity | 4 | 9014 |
| GO:0005215 | transporter activity | 2 | 931 |
| GO:0001071 | transcription factor activity | 3 | 821 |
